# The evolutionary dynamics of the *Helena *retrotransposon revealed by sequenced *Drosophila *genomes

**DOI:** 10.1186/1471-2148-9-174

**Published:** 2009-07-22

**Authors:** Adriana Granzotto, Fabrício R Lopes, Emmanuelle Lerat, Cristina Vieira, Claudia MA Carareto

**Affiliations:** 1 UNESP – São Paulo State University, Laboratory of Molecular Evolution, Department of Biology, 15054-000 São José do Rio Preto, São Paulo, Brazil; 2 Université de Lyon, F-69000, Lyon; Université Lyon 1; CNRS, UMR5558, Laboratoire de Biométrie et Biologie Evolutive, F-69622, Villeurbanne, France

## Abstract

**Background:**

Several studies have shown that genomes contain a mixture of transposable elements, some of which are still active and others ancient relics that have degenerated. This is true for the non-LTR retrotransposon *Helena*, of which only degenerate sequences have been shown to be present in some species (*Drosophila melanogaster*), whereas putatively active sequences are present in others (*D. simulans*). Combining experimental and population analyses with the sequence analysis of the 12 *Drosophila *genomes, we have investigated the evolution of *Helena*, and propose a possible scenario for the evolution of this element.

**Results:**

We show that six species of *Drosophila *have the *Helena *transposable element at different stages of its evolution. The copy number is highly variable among these species, but most of them are truncated at the 5' ends and also harbor several internal deletions and insertions suggesting that they are inactive in all species, except in *D. mojavensis *in which quantitative RT-PCR experiments have identified a putative active copy.

**Conclusion:**

Our data suggest that *Helena *was present in the common ancestor of the *Drosophila *genus, which has been vertically transmitted to the derived lineages, but that it has been lost in some of them. The wide variation in copy number and sequence degeneration in the different species suggest that the evolutionary dynamics of *Helena *depends on the genomic environment of the host species.

## Background

Transposable elements (TEs) are ubiquitous components in prokaryotic and eukaryotic genomes. They constitute the largest part of some of them [1], and play an important role in their evolution [2]. Genome sequencing has shown that TE sequences constitute about 15% of the *Drosophila melanogaster *genome [3,4], about 45% of the human genome [5], and up to 90% of the genomes of some plants [6]. Why species harbor such different proportions of TEs is still unknown, but it may be related to the reproductive characteristics and population size of the host [7,8], and to environmental stresses [9] that may influence TE dynamics. Various different mechanisms may prevent genome invasions by TEs, ranging from DNA deletions [10,11] to epigenetic control mechanisms, such as chromatin conformation [12].

Several studies have shown that genomes harbor a mixture of TEs, some of which are still active, whereas others are ancient relics that have degenerated [13-16]. Degraded elements can result from point mutations or from DNA deletion [17]. Deletions may occur either by recombination, which is common to all classes of elements, or as a consequence of the transposition mechanism of the specific element concerned. For instance, the latter occurs in LINE elements (Long Interspersed Nuclear Elements, also known as non-LTR retrotransposons or retroposons), which are retroelements that use an RNA intermediate to transpose [2]. Their transposition mechanism leads to 5' end truncations of the new inserted sequence. Considerable internal deletions have previously been reported in the inactive copies of several non-LTR retrotransposons [10], and this deletion mechanism seems to act at an high rate, since sequences that are similar at the nucleotide level may have very different internal deletions [10,11,18,19].

One example of this type of TE evolution is the retrotransposon *Helena*, which is a 4,912 bp LINE [18] first reported in the *D. virilis *species [20], and later identified in all the species analyzed in the *melanogaste*r subgroup, as well as in *D. pseudoobscura *[10]. *Helena *has been shown to be present at different stages of its life-cycle in natural populations of *D. melanogaster *and *D. simulans*. Only degenerate copies were found in *D*. *melanogaster *[4,18,19,21,22], whereas in *D. simulans *several different types of sequences have been identified, ranging from highly degenerate to putatively active ones [18]. The analysis of *Helena *in these two closely-related species has shown how important the host genome can be in the evolution of a TE, and how important it is to analyze specific TE families in a wide spectrum of species. This is possible now that the genome sequences of 12 *Drosophila *species [23-26] are available, and *Helena *can be considered to provide an ideal model system for investigating TE evolution across a range of species.

We investigated the evolution of *Helena *sequences using a combination of experimental and population analyses with sequence analyses of the 12 *Drosophila *genomes, and we propose a possible scenario for the evolution of the element in the different host genomic environments that influence the "fate" of TEs.

## Results

### Identification and analysis of reference copies

**Figure 1:**
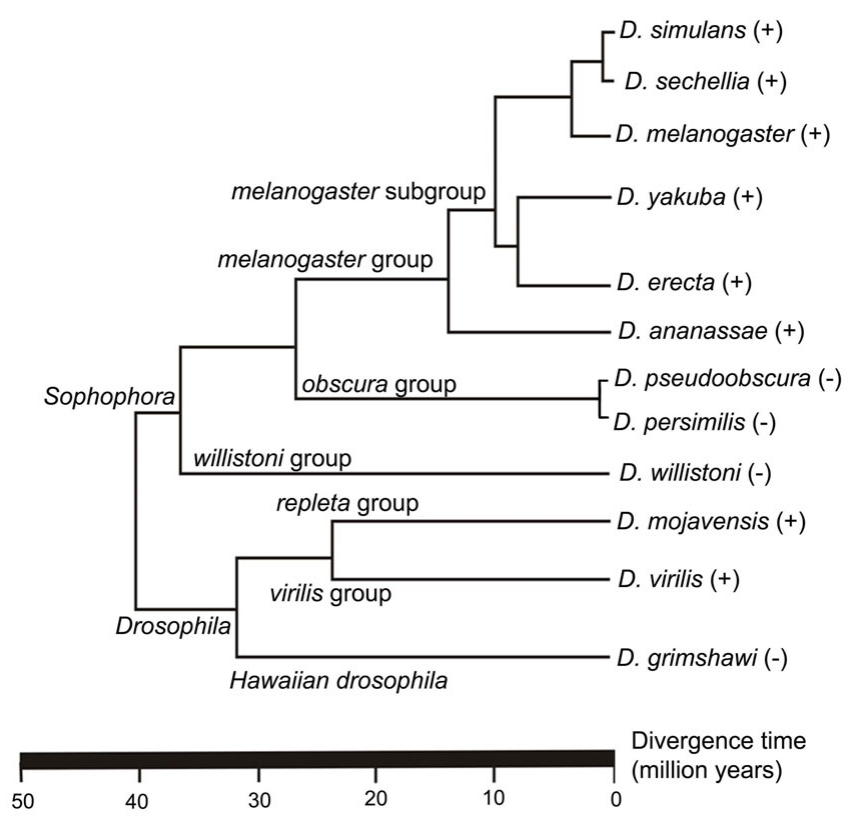
**Phylogenetic tree of the *Drosophila *genus**. Phylogenetic relationships between the *Drosophila *species of which the genomes have been sequenced (modified from http://rana.lbl.gov/drosophila). Presence (+) and absence (-) of *Helena*.

**Figure 2:**
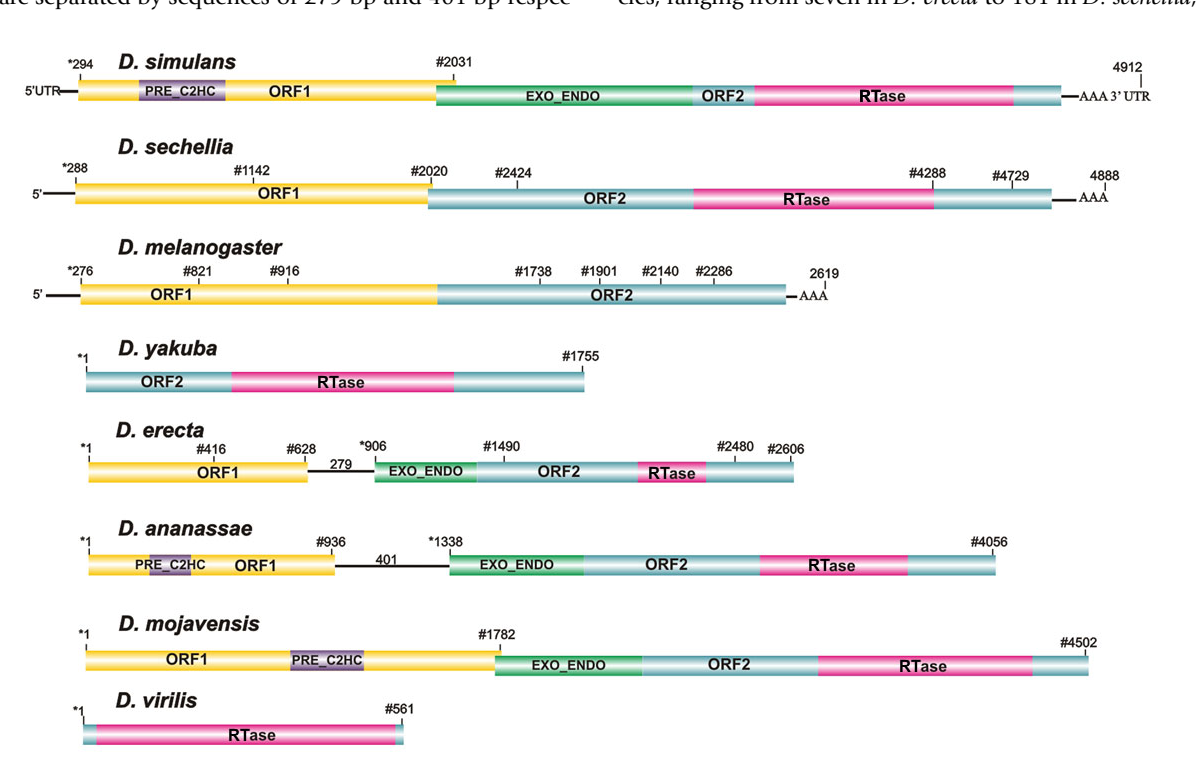
***Helena *structure in *Drosophila *species**. Structure of the reference copies of *Helena*-like elements. DNA sequences: AAA, poly-A tail. Protein sequences: *gag*, nucleocapsid-like domain; *RTase*, reverse transcriptase; *: position of start codons; #: position of stop codons. *D. simulans *and *D. melanogaster *structures are from Rebollo et al. [18]. The sequences are in Additional file 1.

Using the full-length copy of *Helena *already identified in the draft sequence of the *D. simulans *genome [18], and 23 reverse transcriptase (RTase) fragments of the *melanogaster *species group [10], we performed a search for *Helena*-like elements in the other 10 *Drosophila *genomes. We identified *Helena *reference sequences in all the genomes (Additional file 1), apart from *D. pseudoobscura*, *D. persimilis, D. willistoni *and *D. grimshawi *(Figure 1). The results of Blast analyses in *D. pseudoobscura, D. persimilis *and *D. willistoni *revealed only short sequences (10 copies ≤ 210 bp, 23 copies ≤ 251 bp and seven copies ≤ 100 bp, respectively) with percentage identities to the *BS *element that were higher than or similar to those to *Helena *(*D. pseudoobscura *– *BS*: 78%, *Helena*: 69%, *D. persimilis *– *BS*: 80%, *Helena*: 74%, *D. willistoni*: *BS*: 85%, *Helena*: 89%). *BS *is a previously-described LINE element that belongs to the same "jockey clade" as *Helena *[27]. Sequences equally distant from *BS *and *Helena *have also been reported in *D. melanogaster*, and it has suggested that they may constitute a new family, named *Helena*/*BS *[21]. We did not therefore classify these sequences as *Helena *elements, and performed no further analysis of them. Our results for *D. pseudoobscura *are not consistent with previous data reported by Petrov et al. [10], who isolated just one sequence in this species. However, we should note that the same strains were not used in these two studies, and this could explain the differing results. We did not detect any *Helena *sequences in *D. grimshawi*, which is consistent with the fact that this a TE-poor genome [25], but in all the other species the selected reference copies we examined all contained one or two open reading frames (ORF1 and ORF2) (Figure 2).

The *D. sechellia *reference sequence (see Additional File 2 for details) is 4,888 bp long, and contains several of the hallmarks of *Helena*-related elements that have already been characterized: a 15 bp poly-A tail, and two overlapping open reading frames (ORF1 and ORF2). The first ORF is 1,732 bp, and the second 2,870 bp long. Both ORFs are interrupted by three premature stop codons, and the *pol*-like protein contains only the reverse transcriptase domain.

In the other species in the *melanogaster *group, the reference copies are smaller than the *Helena *reference copy in *D. simulans*. In *D. erecta *(see Additional File 3 for details) and *D. ananassae *(Additional File 4), the ORF1 and ORF2 are separated by sequences of 279 bp and 401 bp respectively, but both species contain the apyrimidic endonuclease, the exonuclease and the reverse transcriptase domains (Figure 2). In *D. yakuba *(Additional File 5) and *D. virilis *(Additional File 6) the reference copies consist only of a small ORF2, 1,755 bp and 561 bp in length, respectively.

The reference copy of *D. mojavensis *is the only putatively active *Helena*-like element identified in the species analyzed (Additional File 7). It is 4,502 bp long, and harbors two overlapping ORFs without premature stop codons. The first ORF is 1,782 bp long, and potentially encodes a 593 aa protein. The second is 2,722 bp long, and could encode a 907 aa protein. Moreover, the *gag*-like protein contains a conserved PRE_C_2_HC domain, and the *pol*-like protein contains the three domains required for its function: an apyrimidic endonuclease, an exonuclease and a reverse transcriptase domain. However, the poly-A tail was not identified.

### Genomic Sequence Analyses

**Figure 3:**
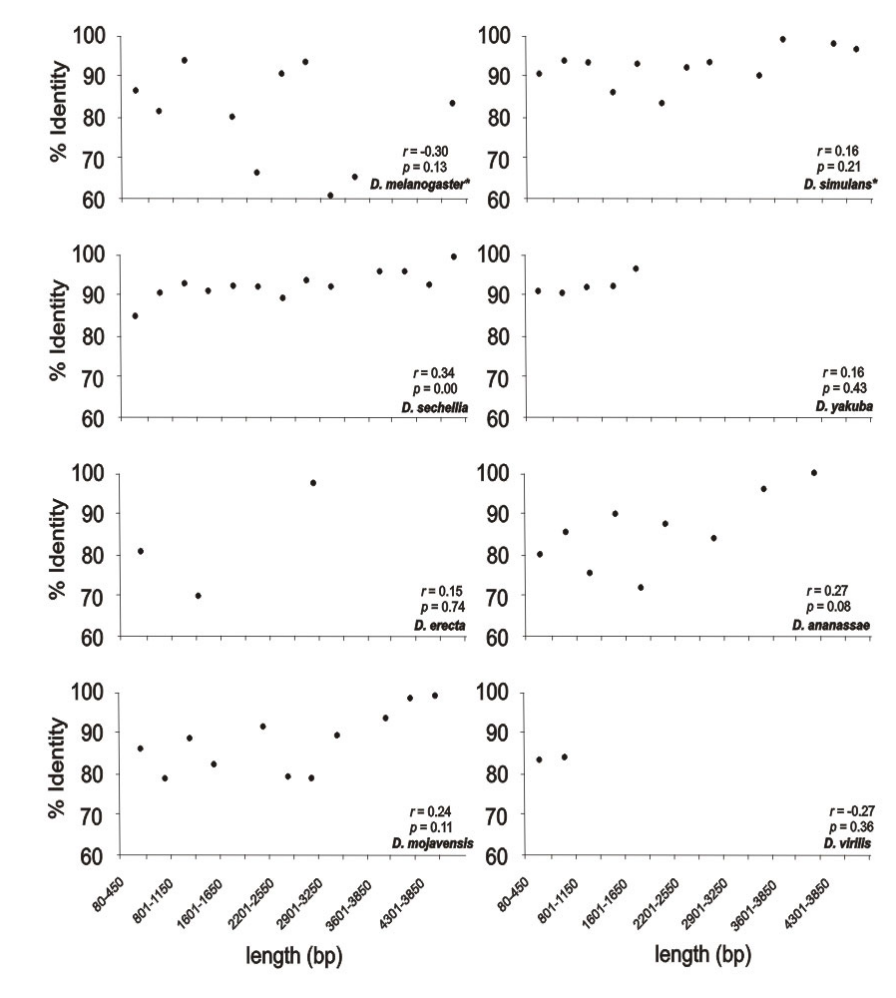
***Helena *copies in different genomes**. Distribution of the lengths (intervals: 350 bp) and percentage identity of the *Helena *copies in *D. simulans, D. melanogaster, D. sechellia, D. yakuba, D. erecta, D. ananassae, D. mojavensis *and *D. virilis*.

The *Helena *copy number for each species is presented in Table 1. This number varies considerably between the species, ranging from seven in *D. erecta *to 181 in *D. sechellia*, with sizes ranging from 80 bp (*D. yakuba*) to 4,888 bp (*D. sechellia*). The average percentage identity of the copies with the reference within each species ranged from 91.51% (*D. ananassae*) to 95.95% (*D. yakuba*). Size-divergence between these copies results from the presence of indels that occur throughout the sequences (Additional File 8). No relationship can be inferred between the size and the percentage identity, since copies with small sizes display an identity level as high as the larger ones (Figure 3). This may be expected when considering only the 5' truncations, produced by the transposition mechanism of *Helena*. However, the different copies reported here are also internally deleted, and can be expected to be old relics of *Helena*, with more neutral mutations than the full-length copies. The only exception concerns *D. sechellia*, in which significant correlation between size of the copies and identity was detected. This is probably due to the large number of copies detected in this species.

**Table 1:** Characterization of the *Helena* elements found in *Drosophila* genomes.

			Length			
				
Species	Size of the reference copy^1 ^(bp)	Number of copies	Min	Max	**Mean **± **SE^2^**	Percentage identity^3^
*D. melanogaster*^4^	4,805	26	91	4,805	1,403 ± 160.0	80.40 ± 1.71
*D. simulans*^4^	4,912	62	107	5,098	1,194 ± 130.0	96.10 ± 0.43
*D. sechellia*	4,888	181	84	4,888	775 ± 73.3	94.11 ± 0.29
*D. yakuba*	1,755	25	80	1,755	712 ± 109.0	95.95 ± 0.68
*D. erecta*	2,606	7	182	2,606	909 ± 358.0	92.85 ± 1.11
*D. ananassae*	4,056	40	83	4,056	812 ± 55.0	91.51 ± 0.74
*D. mojavensis*	4,502	41	105	4,502	1,624 ± 204.0	94.87 ± 1.07
*D. virilis*	561	13	81	569	384 ± 49.3	91.70 ± 1.13

^1^Length of the reference copy;

^2^Mean and standard error of the length between *Helena *copies;

^3^Mean and standard error (in parentheses) of percentage identity between *Helena *copies and the reference sequence

^4^*Helena * copies [18]

Table 2 shows the average GC content for the upstream and downstream regions of the *Helena *insertions for each species. It ranges from 36.8% (*D. erecta*) to 43.1% (*D. melanogaster*). These values are closer to the intergenic values for these species (35.3% – 39.9%) than to those for the gene regions (46.8% – 50.4%), suggesting that *Helena *is more abundant in the non-transcribed genomic environment [4,16,28-30]. The genome of *D. melanogaster *is the only one in which the GC values were intermediate between those of the intergenic and gene regions. This could simply reflect the better annotation available for the *D. melanogaster *genome.

**Table 2:** Average GC content calculated in the first 5,000 nt flanking regions of the *Helena *copies, and of the intergenic and gene (exon plus introns) regions from *Drosophila *genomes

	5,000 bp^1^		Intergenic regions^1^	Gene regions^1^
			
Species	upstream	downstream		
*D. melanogaster*^2^	43.08 ± 0.99	41.76 ± 0.91	38.08 ± 0.06	46.78 ± 0.06
*D. simulans*^2^	40.60 ± 0.54	40.39 ± 0.52	39.92 ± 0.06	50.04 ± 0.05
*D. sechellia*	40.97 ± 0.41	41.02 ± 0.50	39.35 ± 0.05	50.03 ± 0.04
*D. yakuba*	40.43 ± 1.08	41.48 ± 0.83	38.78 ± 0.05	49.99 ± 0.05
*D. erecta*	36.80 ± 1.80	40.75 ± 1.25	39.08 ± 0.05	50.44 ± 0.05
*D. ananassae*	39.83 ± 0.56	39.62 ± 0.59	37.48 ± 0.05	49.44 ± 0.05
*D. mojavensis*	39.44 ± 0.95	37.17 ± 1.11	35.34 ± 0.05	47.81 ± 0.06
*D. virilis*	39.66 ± 0.90	40.00 ± 0. 89	36.52 ± 0.05	48.24 ± 0.05

^1^Mean ± SE;

^2^*Helena *copies [18].

**Figure 4:**
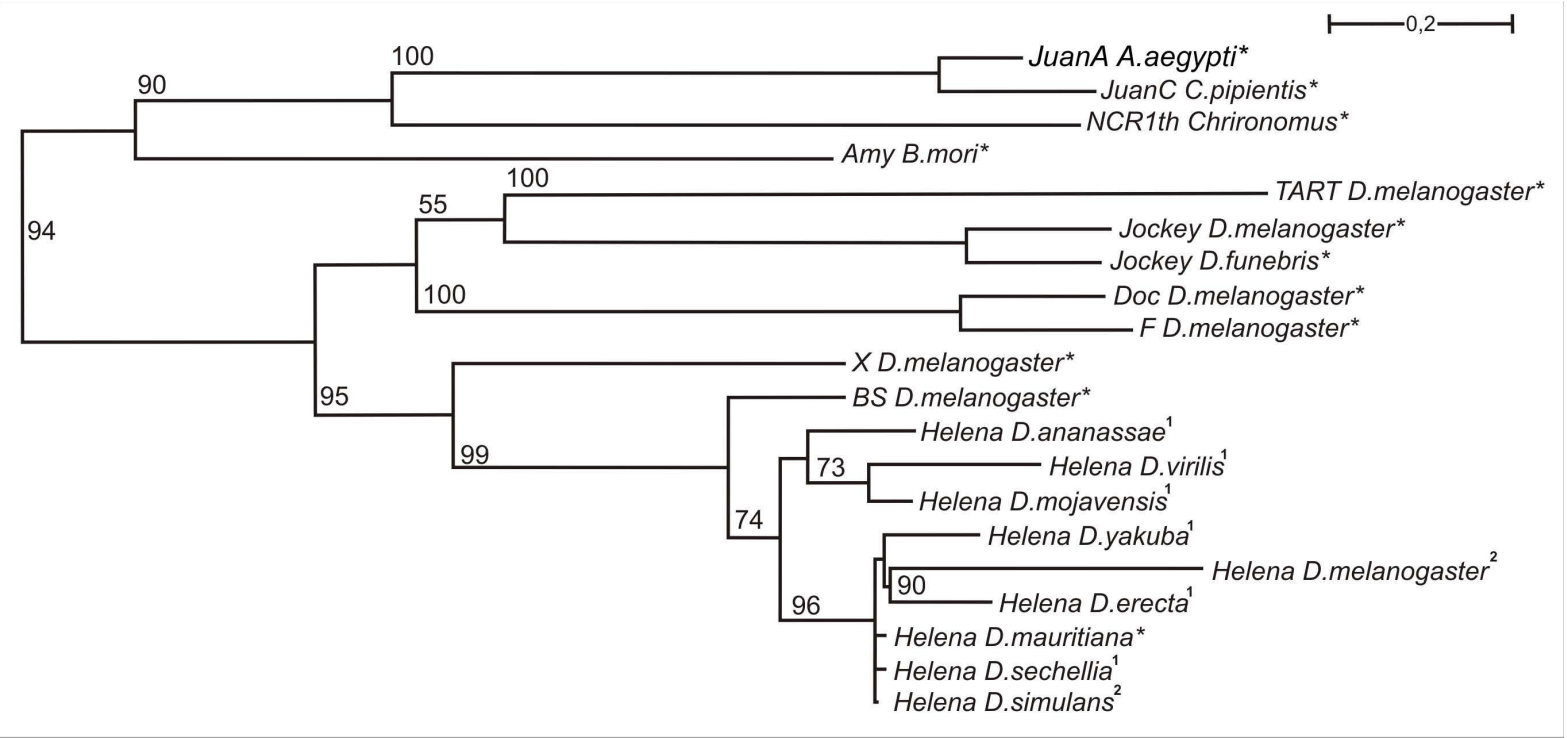
**Phylogenetic tree of RT proteins**. The reconstruction was performed by the maximum likelihood method with the LG model, using the *Helena *reference copies obtained in this study and other *LINE *elements based on their partial reverse transcriptase domains. The sequences used were obtained from GenBank, and are identified by the TE name and the host names (* Sequences obtained from GenBank; ^1^Sequences obtained in this study; ^2^*Helena *sequences obtained by Rebollo et al. [18)]. The numbers indicate the branch support calculated by bootstrap analysis consisting of 100 replicates. Only bootstrap values greater than 50% are indicated.

*Helena*-related sequences form a monophyletic clade (Figure 4) that includes a well-defined cluster of the species of the *melanogaster *subgroup (*D. yakuba*, *D. simulans*, *D. melanogaster*, *D. erecta *and *D. sechellia*). The other group contained *D. ananassae*, *D. virilis *and *D. mojavensis*. This phylogeny is typical for a TE with vertical transmission.

### Analysis of the activity in natural populations

**Figure 5:**
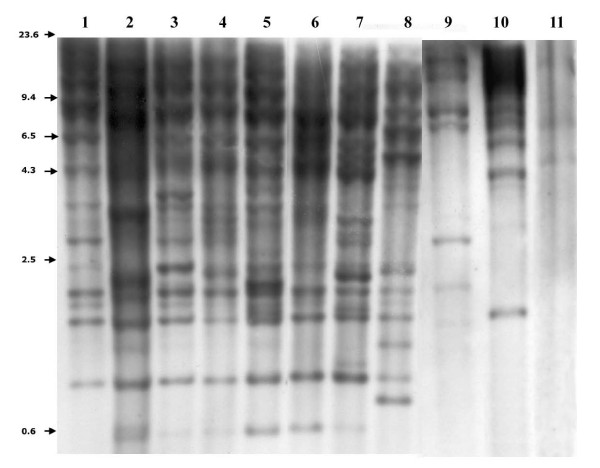
**Southern blot analysis of *Helena *in *D*. *simulans *and *D. mojavensis *populations**. Lanes 1 to 8 are *D. simulans *populations (1: North America (Tucson stock center: 14021-0251.195), 2: Junco do Serido (PB, Brazil), 3: Itaúnas (ES, Brazil), 4: Lençóis (BA, Brazil), 5: Onda Verde (SP, Brazil), 6: Ratones (SC, Brazil), 7: Seychelles (Seychelles), 8: New Caledonia (Tucson stock center: 14021-0251.216). Lanes 9–11 are *D. mojavensis *populations (9: Catalina Island (California, U.S.A, Tucson stock center 15081-1352.02), 10: Grand Canyon (Arizona, U.S.A), 11: Sonora (Mexico, Tucson stock center: 15081-1352.24)).

**Figure 6:**
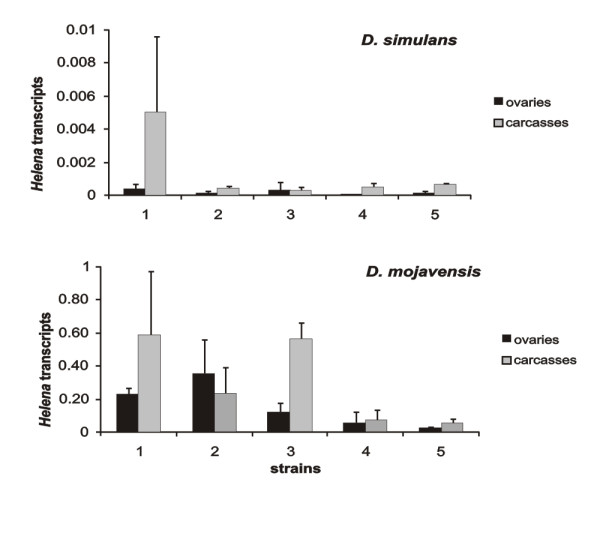
**Analysis of *Helena *activity in natural populations**. *Helena *transcripts (ratio *Helena*/*rp49*) of ovaries and carcasses of *D. simulans *and *D. mojavensis *(see Methods for additional information). *D. simulans s*trains from the *Drosophila *species stock center: 1 (14021-0251.195 – North America, U.S.A), 2 (14021-0251.194 – Winters, California, U.S.A), 3 (14021-0251.198 – Noumea, New Caledonia), and from natural populations 4 (Amieu, France), 5 (Valence, France). *D. mojavensis *strains from natural populations: 1 (Grand Canyon, Arizona, U.S.A), and from the *Drosophila *species stock center 2 (15081-1352.02 – Catalina Island, California, U.S.A), 3 (15081-1352.09 – Santa Rosa Mountains, Arizona, U.S.A), 4 (15081-1352.24 – Sonora, Mexico), 5 (15081-1352.22 – Catalina Island, California, U.S.A). Black = ovaries. Gray = Carcasses. Standard deviation is indicated with bars.

Since our *in-silico *analyses show that only *D. simulans *and *D. mojavensis *harbor full-length, putatively active copies, we performed Southern blot analyses on several populations of these two species to infer their TE activity (Figure 5 and Additional File 9). Our results show that these species display insertion polymorphism, suggesting that *Helena *is active. We cannot of course exclude the possibility of restriction fragment length polymorphism, which would lead to the same kind of result. The qRT-PCR experiments on these two species show that *Helena *is highly expressed in *D. mojavensis*, which displayed population variability that contrasted with that of *D. simulans*, in which the levels of expression were much lower (Figure 6). We were only able to compare the *Helena *transcripts, because the housekeeping gene (*rp49*) used to normalize qRT-PCR is equally expressed in both species (Additional File 10).

## Discussion

This study describes the evolutionary dynamics of the *Helena *non-LTR retrotransposon in the sequenced *Drosophila *genomes. We have shown that *Helena *occurs in *D. sechellia*, *D. yakuba*, *D. erecta *and *D. ananassae *(*melanogaster *species group), in *D. mojavensis *(*repleta *group) and in *D. virilis *(*virilis *group), as well as in *D. melanogaster *and *D. simulans*, which had been studied previously [10,18]. In *D. pseudoobscura*, *D. persimilis *and *D. willistoni*, the small copies that we found displayed similar percentage identities to *Helena *as to *BS*, a non-LTR retrotransposon related to *Helena*. Sequences with the same kind of similarity have been reported in *D. melanogaster*, and were grouped by the authors as *Helena/BS *family [21]. The *D. pseudoobscura*, *D. persimilis *and *D. willistoni *sequences we found can be included in this family. We agree with the suggestion that there could be members of the *Helena/BS *family in these species as shown in *D. melanogaster *[21], and so we did not include them in our study. The 907 bp sequence of *Helena *that had been previously described (GenBank AF012036) in a strain (stock center: 14011-0121-0, Tucson, Arizona) of *D. pseudoobscura *[10] was not found in the sequenced genome of this species. Our phylogenetic analysis shows that *Helena *is a monophyletic group of sequences patchily distributed in the species of the two subgenera of the genus *Drosophila *(Figure 1). Taking our data together with those from other authors [10,18,20], we can conclude that *Helena *was present in the common ancestor of the *Drosophila *genus, and has been vertically transmitted to the derived lineages, but subsequently lost in some of them, or at least diverged so much as to escape detection. The high variability in copy number and sequence degeneration in the different species shows that the evolutionary dynamics of *Helena *depends on the genomic environment, as has already been reported for other retrotransposons, such as *Tirant *[31].

Recent work has reported the presence of deteriorated and inactivated *Helena *in the *D*. *melanogaster *genome, but also of a full-length copy in *D. simulans *carrying all the structures required for activity, and with a high level of insertion polymorphism in the natural populations [18]. This suggests that in this species *Helena *is either still active or has been active until recently. In our analysis, all the reference copies in the other 10 *Drosophila *genomes were found to be devoid of intact ORFs, except in *D. mojavensis*. In this species, two copies of *Helena *contain intact ORFs, which suggest that full-length and potentially active *Helena *sequences could exist. This means that this species may offer a unique opportunity for studying the evolutionary dynamics of *Helena*.

Our analysis of the copy numbers reveals a strikingly variable distribution of *Helena *in the 12 species. In *D. sechellia*, 181 copies of *Helena *were observed, whereas in *D. erecta *only seven copies were identified. Even though these genomes have not been well annotated, and there are some low quality sequences that could bias copy number estimation, this should not invalidate the tendency identified. It has been proposed that effective population size could be one of the main factors accounting for differences in copy number of TEs [32,33], with selection against TEs being less effective in smaller populations [34]. This seems to apply to *D. sechellia*. This species is restricted to the Seychelles Islands in the Indian Ocean, and is the most specialized fly within the *melanogaster *group. It has a very low effective population size [35,36], and carries the highest number of *Helena *sequences recorded (181 copies). We would expect to find a similar scenario for *D*.*erecta*, which is also a specialized species with a small population size [37,38]. However, as has also been observed for the *mariner *element, the observed copy number of *Helena *is not in fact consistent with this hypothesis [37]. This means that other factors may be affecting the copy number of TEs, including genomic and environmental features.

We did not observe any major differences in copy number between the two species of the *Drosophila *subgenus, even though *D. mojavensis *has a higher copy number than *D. virilis*. What is more striking is that the only potentially full-length active copies identified were detected in *D. mojavensis*, together with the other potentially active copy previously described in *D. simulans *[18]. The Southern blot analyses of several natural populations of *D. mojavensis *and *D. simulans *suggest that *Helena *has recently displayed transpositional activity. However, we confirmed that the transcriptional activity is low in *D. simulans*, as had previously been suggested [18], and this clearly indicates that *Helena *is being lost in this species. In *D. mojavensis*, the situation is completely different. We have observed high levels of *Helena *transcripts in several populations, confirming its activity. Moreover, expression varied between different populations, indicating that *Helena *must be present at different stages of its evolutionary cycle, i.e. different stages of activity, in different populations within this species.

Our analysis has shown that the evolution of *Helena *is influenced by the host species, resulting in differences in copy number, degradation and activity. In all the species analyzed, *D. mojavensis *is the only one in which the *Helena *has survived, which gives us a unique opportunity to observe the "fate" of this TE. However, it is crucial to investigate natural populations from this and other species in the *repleta *group in order to find out how widespread the scenario described here actually is, and to understand the process and speed of the degradation and extinction of *Helena*.

## Conclusion

Here we show that six species of *Drosophila *have *Helena *transposable elements at different stages of its evolution, and may represent different stages of the TE evolutionary cycle. The copy number is highly variable in different species, but most of them are truncated at the 5' ends and display several internal deletions and insertions. In all the species analyzed, *Helena *has only survived in *D. mojavensis*, and this gives us a unique opportunity to track the "fate" of this TE.

## Methods

### In-silico analyses

The draft sequence data from the 10 related *Drosophila *Genome Sequencing Projects used in this study are listed in the Table 3. Sequence searches for the *Helena *element were carried out using the 4,912 bp full-length sequence of *Helena *characterized in *D. simulans *[18] as the query. Twenty-three partial sequences of reverse transcriptases (RTases) of *Helena *from the *melanogaster *group [10], and the two LINE elements closest to *Helena*, *BS *[27] and *X *[39] were found. Comparisons between the query and the *Drosophila *data set were performed using High Scoring Pairs (HSPs) within BLAST algorithms [40] with an E-value ≤ 1e^-10^. The sequences correspond to the three best BLAST hits, which were more than 80 bp in length, and were selected and extended to include 3 kb of the flanking regions. These sequences were analyzed using the ORF-FINDER program to identify putative occurrences of coding regions [41]. Conserved domains were predicted using the "Conserved domain search" tool from NCBI, as well as BLASTn and BLASTx searches against the nt and nr databases, respectively. Thus, we determined the most complete *Helena *sequence in each of the 10 *Drosophila *species to be considered as a reference copy within each genome. To determine the copy number in each species, the previously-determined reference copy was blasted against the full genomes. Significant matches were required to be more than 80 bp long, and to have at least 80% identity [42]. Regions similar to *Helena *that were separated by less than 200 bp were considered to be a single insertion. For each genome, the copies have been aligned with the reference, using MUSCLE [43] and the % identity to the reference was computed using the DNADIST program from the PHYLIP package [44]. The flanking regions of each insertion were extracted for analysis of the GC content in the first 5,000 nt using "geecee" of the EMBOSS package [45]. The GC contents of the flanking regions of the *Helena *elements were compared to those of the intergenic regions and genes (exon plus introns) in the other *Drosophila *genome versions, as noted in the Table 3.

**Table 3:** Genome sequences used in this study

Genomes	Genome version used for *Helena *identification	Genome version used for other analyses^5^
*D. melanogaster*	BDGP release 4^1^	Release 5.5
*D. simulans*	WUSTL mosaic^2^	Release 1.0
*D. sechellia*	CAF1^3^	Release 1.0
*D. yakuba*	CAF1^3^	Release 1.0
*D. erecta*	CAF1^3^	Release 1.0
*D. ananassae*	CAF1^3^	Release 1.0
*D. pseudoobscura*	Release 2.0^4^	Release 2.0
*D. persimilis*	CAF1^3^	Release 1.0
*D. willistoni*	CAF1^3^	Release 1.0
*D. mojavensis*	CAF1^3^	Release 1.0
*D. virilis*	CAF1^3^	Release 1.0
*D. grimshawi*	CAF1^3^	Release 1.0

^1^Flybase [50];

^2^Genome Sequencing Center at the Washington University at St. Louis [51];

^3^AAA of 12 related *Drosophila *species [52];

^4 ^Flybase [50];

^5^Flybase [50].

### Phylogenetic analyses

The sequences used in the evolutionary analysis were obtained from GenBank: *Jockey *(M22874), *TART *(U14101), *Doc *(X17551), *F *(M17214), *BS *(X77571) and *X *(AF237761) of *D. melanogaster; Jockey *of *D. funebris *(M38437); *Amy *of *Bombyx mori *(U07847); *JuanA *of *Aedes aegypti *(M95171); *JuanC *of *Culens pipientis *(M91082); *NCR1th *of *Chironomus tentans *(L79944); *Helena *of *D. mauritiana *(AF012043), *D. simulans *and *D. melanogaster *[18]. The multiple alignment of the RTase proteins from 11 *LINEs *of the *Jockey *clade, and the six *Helena *reference sequences described here (see Additional Files 1, 2, 3, 4, 5 and 6 for details) was performed using CLUSTALW [46] with the default parameters, and the alignment was manually curated using a sequence editor. The evolutionary relationships were reconstructed using the maximum likelihood method for the LG model [47] as implemented in the PhyML software [48]. The bootstrap analysis consisted of 100 replicates.

### Southern blot

The occurrence of *Helena *in populations of *D. simulans *and *D. mojavensis *(Additional File 8) was confirmed by Southern blot using the detection system *Gene Images *CDP-Star detection module (Amersham Biosciences, Little Chalfont, UK). Genomic DNA was prepared from 50 adult flies [49] and digested by *Hind *III, which has no restriction site within the *Helena *sequence, so that each hybridized fragment would correspond to a single genomic insertion. The restricted fragments were separated in 1% agarose gels, and transferred to Hybond N+ membranes (Amersham Biosciences, Little Chalfont, UK). Blots were prehybridized for 1 h at 60°C in 5× SSC, in 5% dextran sulfate, subjected to 20-fold dilution of the liquid block, and hybridized overnight with the probes. Blots were washed twice with 0.2× SSC, 0.5% SDS, and then exposed to autoradiographic film for 20 minutes at room temperature. A 644 bp *Helena *sequence amplified from plasmid AF012044 (DsechF: 5' AGGATTTGTCATGCCACGCT 3' e DsechR R 5' TGTTTGGTGCTGCCATGTGT 3'), and a 674 bp sequence, corresponding to the RTase of *D. mojavensis Helena* (DmojF: 5' TAAGAGGCCATAGTACGGAGCAGGTA3' and DmojR: 5' GCGAATTGGAACAGGCTAACGCAT 3'), were used as probes for the *D. simulans *and *D. mojavensis *populations, respectively.

### Quantitative RT-PCR (qRT-PCR)

The expression profiles of *Helena *RTase in different populations of *D. simulans *and *D. mojavensis *(Additional File 8) were determined by real-time PCR. For this analysis, 20 ovaries and 15 carcasses of each population were used to extract total RNA using RNeasy kit (Qiagen). 1 μg of total RNA treated with DNAse Ambion was converted into cDNA using Thermoscript kit (Invitrogen) primed with oligo-dt and random primers mix. The cDNA samples were diluted 50 fold, and PCR was carried out using QuantiTect SYBR Green PCR kit (Roche) on the LightCycler (Roche) using primers specific to the *Helena *RTase of *D. simulans *(RTase_D.simF: 5'ACAGCAGAGAGACAGCTAACGGAC 3', Rtase_DsimR: 5' AGATGTGTTGCTTGCAGGGTCTGA 3' and *D. mojavensis *(RTase_DmojF: 5' TTGGTCCGCTGCTGTTCTCCTT 3', Rtase_DmojR: 5' TGAGATTCCACCGCTTGCACCA 3') that amplify 193 bp and 204 bp respectively. Quantitative PCR cycling conditions were 5 min at 95°C (1 cycle), 15 s at 95°C, followed by 10 s at 62°C and 20 s at 72°C (50 cycles). A negative control for DNA contamination of cDNA of each population (without Thermoscript enzyme) was tested (data not shown). Reactions were done in duplicate, and standard curves were calculated from serial dilutions of specific amplified PCR fragments. The quantity of the transcripts was estimated relative to the RP49 expression (qPCR fragments of 182 bp and 167 bp for *D. simulans *and *D. mojavensis *respectively). Primers were: RP49_DsimF: 5' CGGATCGATATGCTAAGCTGT 3', RP49_DsimR: 5' GCGCTTGTTCGATCCGTA 3', RP49_DmojF: 5' GTCGTCGCTTCAAGGGCCAAT 3', RP49_DmojR: 5' ATGGGCGATCTCACCGCAGTA 3'. In both species, RP49 expression is equivalent (Additional Figure nine) allowing relative expression of *Helena *to be comparable between *D. mojavensis *and *D. simulans*. Hence, *Helena *transcripts plotted in Figure 6 are the result of quantification of *Helena *transcripts normalized by the quantification of RP49 transcripts for each strain.

## Abbreviations

DOA: dead on arrival; LINE: long interspersed nuclear element; LTR: long terminal repeat; ORF: open reading frame; TE: transposable element; RTase: reverse transcriptase.

## Authors' contributions

AG carried out the molecular and genetic studies, AG, FRL and EL did the bioinformatic analyses, CV and CMAC designed and coordinated the study. All the authors contributed to data analyses and the writing of the paper. All the authors have read and approved the final manuscript.

## Supplementary Material

Additional file 1 **Sequences of the *Helena *reference copies**. *Helena *sequences used to determine the structure of the element, for each species. Click here for file

Additional file 2 ***Helena *copies in the *Drosophila sechellia *sequenced genome**. The data provided is a list of *D. sechellia *copies. Click here for file

Additional file 3 ***Helena *copies in the *Drosophila erecta *sequenced genome**. The data provided is a list of *D. erecta *copies. Click here for file

Additional file 4 ***Helena *copies in the *Drosophila ananassae *sequenced genome**. The data provided is a list of *D. ananassae *copies. Click here for file

Additional file 5 ***Helena *copies in the *Drosophila yakuba *sequenced genome**. The data provided is a list of *D. yakuba *copies. Click here for file

Additional file 6 ***Helena *copies in the *Drosophila virilis *sequenced genome**. The data provided is a list of *D. virilis *copies. Click here for file

Additional file 7 ***Helena *copies in the *Drosophila mojavensis *sequenced genome**. The data provided is a list of *D. mojavensis*. Click here for file

Additional file 8 **Schematic representation of *Helena***. Schematic representation of *Helena *copies in *D. sechellia, D. yakuba, D. erecta, D. ananassae, D. mojavensis *and *D. virilis*. The sequences represented have at least 90% identity and 50% of the length of the reference copy, and with e-values of less than 10e^-10^. Spaces = indels. The first schematic representation is the reference copy in each species. White = gag. Gray = RTase. Click here for file

Additional file 9 **Species and strains, geographic origin and year of collection**. The data provided is a list of *D. simulans *and *D. mojavensis *used in this study for analysis of *Helena *activity in natural populations. Click here for file

Additional file 10 **Mean CT of the *rp49* control gene and *Helena***. Ct comparison between *rp49 *(reference) and *Helena *real time PCR assays of *D. simulans *and *D. mojavensis*. Gray = mean CT of *D. simulans*. Black = mean CT of *D. mojavensis*. Std = standard deviation. Click here for file
