# Supplementary material for: The evolutionary dynamics of the Helena retrotransposon revealed by sequenced Drosophila genomes
Source: BMC Evol Biol. 2009 Jul 22;9:174. doi: 10.1186/1471-2148-9-174 (PMC3087515; doi:10.1186/1471-2148-9-174)
Supplement: Additional file 1 — Sequences of the Helena reference copies. Helena sequences used to determine the structure of the element, for each species. [file 1471-2148-9-174-S1.pdf]

Additional File 1. Sequences of the *Helena* reference copies.

>*Helena_D.sechellia*_super_107_28043_32931
TGAGCTGGTCACACTGAGCGGTTGTTTATAGTTTTTGTCTGTGAATTTCACTTTAAATTTAATTCGCAAA
CCTGCATTCGTAGCTGTGCAAAACAGCACGCATTCTAAAATTTGGACTCTGCCTTGCAGAGTCGGCATGT
GAACATCAACAAAGATCGGTGGATAAGGTGCGAAAAAGTTGCATTCTCTGCCCATTGGGCCCCTCACCAC
GTGGCAGCCGCACAAGGGAGGCAACAAGCAACGTACTTATTAGAAAAAAATAAATAGACCGGATTAGTAA
CTGTAAAATGGATACGAACAACGAAACTTCTAACTCTCAGCTTAGAAATAAGCCGATTGATTATGAAGAA
ATGCGGCAAGTAGCTGGTAAGTCTCCACTGGATATCAGGATGGAGTTATTGAATCAACGCTCTTCATGCA
CTGAATCTGTTAACCTAAGCACAATAACTACGACGATAACATTTACAAGTGCTACTTGCAATACTACATC
TACAACAACTGCAGCAATCAGTAGGCCGAGCTCCAGAGAAAAAGCTGCTACTAGATTGTCTGATGCGCAA
AAACTTCTGACGAAAGAGAGGAAGAGGAAGACCAATTTAACACCCAACCATAGCTCTAAACGAGCTGTCA
GAGACGCGAAGCTGGAGCCATATCCAGCCATAAATAGTAATTCAAACTCAAACAATAGGTTTGCCATGCT
AGACATGGAATTGGACGAAACCAGTGATGGCATAGAAACTCCACAAGTTTGTCATGATGTCGCATTCACC
TCGTGTGCATCGGCTGCTCCGAATATTCCTAATGATGACTGTGTACCAAATGTGACATCCAATAGCCCAC
AACAGTATACTGATAAGCAGAATTCTTATAAACAATCAAAACCACCGCAAATAGTACTGAGCCTTACCAA
TCTTAATGATCTCTATGAGCTCATTACGGAGGTCACTAGCCTAGATAATTTAACAGTTAAAGTCAATCAA
GGGGAAACAGTGAGAATATTACCCAAAGACTCTGATACTTACAGAGCCATTATTAATATTTTTGATAATT
CGGGAATTGAATTCCATACGTACCAAATGAAGGAAGAGAAGCCTCACAGAATAGTTGTTAACCCTAACCT
ACGAAATTATCGACAACTTTAAAACATATGGCTTTGATGTTCTACAAGTACACAACCCAAGATCCAGGAG
AAATAGAGAAGAAAAACTTAATATATTCTTCATTAATATAAACCCTTTGTGCAAAAATTAATGACATATA
CGATATTAACACAGTATGCCGACAGAAAGTGCGGATAGAAAGAATGCGTAAATCATCTGAAATTGCTCAA
TGCATACGTTGTCAGGAATTCGGCCACACAGCTAAATACTGTCGTCGTCATCCCAACTGTGCTCGATGTG
GTGAAAATCACTTAACCAAGCTATGCGTACTTCCCAATGATCAACAGCCTATCTGTATACACTGTGGAGG
AAATCACACGGCAAGTTACAAGGGTTGCCAGTTTTACCAGGAGTATCTTCGACGATCATTGGGCACTGTA
AAGAAGCAACAACCTAAGCAAGCCAGGTTTGATAAAAGTGCAACAAACCAACAACAACCCCAGCAAAAAC
AGCAGCATGCAATAGCTAGCACTCCCAAAGATCATACTGGGAGCTTGTCCTACGCAGATATTGCAAAAAA
TGGCAATACAACAGCCCAGCCTCGTCTACATAATGTACAATTAAAGGGAACTAATATTAAGCAGCAACAC
CCGCTTGACGTTCAGTCAATATTGGCACAGCAACAGGAACAATTTATGAAGTGGCAGCAACAGCTTCAAC
AGCAACAACAGCAGCAATTCCTATCGTGGCTACTACAGCAGCAACAGGAGCAACAACAACACAACAAGTT
GAATAGTCAACGACTCGAAAGGCTGGAAAAAATTGTTTTTGAAATGGCCAATATGCTGAAGCAACGGACT
GGGGATACATCGGCTCCCCAACTCCATAGTAACGATTCACCATCGCAATTAACCCTCTGAAGATTCTTAT
CTGGAATGTAAATGGTTTTTCAGGTAAAGCCAGAGAAGTCGAGCTCTTCGCACACAACAACGGCATTGAC
ATTCTTCTCCTAAACGAGATCAGACTCAACAGAGGGAACACAGTTAAGATATATGGGTATAGCTTTTATC
CCGCATACAAACCTTCAAGCCATAATCACGGAATGGGAGGAGCAGCAGTACTGGTGAGAAGCTCTCTTCG
TCATTTCCCGCAAAGAGTTATTGAAACGAGAACTATTCAGATGTCTTCAGTCAAGGTCTCCACCGGGCTG
GGAGATATGGAATTTAGCGCGATTTACTGTCCACCAACAAATAGAATTGAGGAAAGGCACTTCAGTGACA
TACTTGTCTCTTGTGGACAAAGGTATTTCGTTGGTGGGGACTGAAACGCCCGATATTGGCTATGGGGTGA
TACGTACAATTAACCCAGAGGTCGAGAACTAGCAGAAGCCATTTCAGCCAGAGGTGCTTATATCCTTGCA
ACAGGTTCACCAACTAGATACCCACATGTGCCCAGTCACAGACCTACCTGCATTGATTTTGCTGTGTACC
ATGGGATAAACTTAGACAGAACTAGTATTTCTGAAAATTGGGATCTAGACTCCGATCATGTAGCCCTTGT
GGCTACTGTACAAACAGAAGGTGCCTATGTTAGACCATGCTCTCGGTTAATAAACAGCCGAACTGATCTC
CTTGTTTTCAGACAACATCTGGAAAACTCTCTCCAATTAAATACGGTTCTGAGCTCTAAGGAAGATATCG
AGAACGCAGTCTAAGAACGCAGTCTAACGCAAAATATATATATAGCCGCTTCTGCTTCTACGCCCGAGAT
ACGCCCCAGAAGTTATGGTATTGTTCTAACAAGAGAGGCCAGATAACTTATCAGAACTAAGAGACGCCTT
CGAAGAAGAGCAATTCGAACTCAGGATCCATGGGACAGAATCTTGTGGAACCGAGCAGCAAAGCAACTCA
AAGTCATCTTAAGGGAACTCAGAAGTGATTTCTTTGAGCAAAAATTATCCTCCATGGACTACACCGTTGA
TGCAAACTATTCGCTGTGGAAGTGCACAAAAGCGCTTAAGCGACAACCACTTCGATGGGTACCCGTACGC
TGTCCAGGTGGGGAATTTGCAAAAACTTGAAGTGGAACAGGCTAATGCATTCGGCTTCCACCTAGAGTAT
CGCTTCACTCCTTACGACTTCGCCACGACAGAACAAATAAGAGAGACTCACCAGTACCTACAAATGCCAT
TGCAGATGTCTTGGCCTATTAAGCCAATAAGGATAGAAGAAATCCTTGAAATAATTAAATTACTGCCGAA
GCATAAAGCTCCAGGAATTGACAGGATTTGTCATGCCACGCTAAAGGTTTTACCTATAAAAGCGATAATA
TATATAGCACTAATCTTTAATGCTATTTTAAGGATCCAAGTGTTCCCAAGACAGTGGAAAATGCCTGCTA
TTTTGATGATCCACAAGCCTGGAAAGCCGGAAGATGATCCAGAGTCGTATCGGCCTATAAGCCTCTTACC
CTCACTTTCTAAATTATGGGAGAGACTTATTGCCAATCGGATAAACGACATTATAAGACAAGGCAATATC
TTGCCGGATCATCAATTTGGATTTCGAAAGGGACACGGAACTATTGAACAGGTCCACAGACTGGTGAAAC
ACATACTACAGGCTTTTGACGACTGCGAGTACTCCAACGCCGTCTTTATAGATATGCAACAAGCCTTCGA
CAAAGTATGGCATGTTGGATTATTATGCAAGATAAAGACCCTTCTACCTGCGCCCTACTTCTGTATTTTA
AAGTCATATCTAGAAGAAAGAGAATTTAAAATCACGGTGAGAAATAGCTACTCCTCTATATACCCAATGA
GAGCTGGAGTCCCTCAGGGCAGTGTTCTCGGACCGCTACTGTATTCCTTGTACACTGCTGATATCCCTTG
CCCGAGTTTCGAACACATGGCAGCACCGAACAGGACTCTTATTGCAACCTATGCAGATGACATCGCAGTT
GTATATAACTCTAGGGACAGCAGAGAGACAGCTAACGGACTACAAGAATATATTAATGATCTGGCAGCCT
GGTGTAAACGGTGGAACCTAAAAATAAACCCACTGAAAACAACAAACCCGTGCTTCACGTTAAAAACGCT
TATCCCGAACACCCCTCCAATTCGGCTAGAAGGAGTTACCCTGAATCAGACCCTGCAAGCAACACATCTA
GGTATCACCCTGGATTAACGGCTCACCTTTGGGCCGCATCTAAAAAAAACAGTAAAAAAATGTGGCCACA
GATTACAACAGCTGAGATGGCACATGAATAGAAGGAGCACTCTTTCGATGAGGTGCAAAAGAGCTGTGTA
TGCGCACTGTATCGTACCGATATGGTTATACGGGATCCAGATTTGAGGAATTGCAGCCAAATCGAATTAT
AATCGTATCCAGGTGATGCAAAATCGCGCATTACGACAAATAACCAACTGCCCCTGGTATGTACCTAACT
CTACACTCCATAAAGACCTCAATATTCACACAGTTGAGTCACAAATTGGGAGACATACATGTCGATATAG

TGACAGATTACTGAGCCATAGCAGTCTTCTTGCAAGACGTCTCATCCCCGCTCGACCTCTGAGAAGACTT
AAACGGCAAGGCTTCGCCAAGCCAATTGGGCAGCAGTAAAAACTCTTCATTTATGTTCCTACTCCACTTA
TTTTATTTTATAGTGTTTGGTAATTGATAAGAATATTTCTCCACACTGTGATGTTAAGATACGATATTAT
GATGTACGGATTCTTCACTTAATAAATATAAAAAAAAAAAAAAAAGTTCGTTAAAAACA

>*Helena_D.yakuba*_contig197.8_2845_4599
CCATGGGACAGAATCTTGTGGAACCGAGCAGCAAAGCAACTGAAAGGCATTCTAAGGGAGCTCAGAAATG
ATTTCTTTGAGCAAAAATTATCTTCCATGGACTACACAGTTGATGCAAACTATTCGCTGTGGAAGTGCAC
AAAAGCGCTCAAGAGACAACCATTTCGATGGGTACCAGTACGATGTCCTGGCGGGCAAATTGCAAAAAGT
GAAGTGGAACAGGCTAATGCATTCGGCTACCACCTTGAGGATCATTTCACTCCTTATGACTTCGCCACGA
CAGAGCAGATAAGTGAGACTCACCAGTACCTACAAATGCCATTGCAGATGTCTTTGCCTATTAAGCCAAT
AAGAATAGAAGAAATATCTGAAGTAATTAAATCACTGCCGAAACATAAAGCTCCAGGATATGACAGGATC
TGCCACGCCACGCTAAAGGTTTTACCTATAAAAGCGATCATCTATATAGCACTTATCTTTAACGCTATTT
TAAGGGTCCAAGTATTCCCAAGACAGTGGAAAATGGCTGTTATCATGATGATCCACAAACCCGGGAAACC
AGAAGCTGATCCTGAGTCTTATCGGCCTATAAGCCTGTTACCCTCCCTCTCTAAATTATGGGAAAGACTT
CTTGTCAAAAGGATCAATGACATTGTAAGACAAGGTTATATCTTGCCGGACCATCAATTTGGATTTCGGA
AGGGACACGGCACTGTAGAACAGGTCCACAGACTGGTGAAGCACATATTACAGGCTTTTGACGACTGCGA
ATACGCAAACGCTGTCTTTATAGATATGCAACAAGCCTTCGACAAAGTATGGCATGTTGGATTATTATGC
AAGATAAAGACCATGCTTCCTGCGCCCTACTTCTGTATTTTAAAGTCATATCTAGAGGGACGAGAATTTA
AAATCACAGTTAGGAATAACTACTCTACCGTATATCCAATGAGAGCTGGAGTCCCACAGGGCAGCGTCCT
TGGACCACTACTATACTCCTTGTACACTGCAGATATCCCTTGCCCGAACTTCGATCACATGGAAGCACCC
TACAAGGCTCTTATGGCAACCTATGCAGATGACATCGCAGTGGTGTATAACTCTGGGGACAGCGGAGAGG
CAGCGAAAGGACTGCAAGAATACATTAATGCTCTAGCAGCCTGGTGTAAACGGTGGAATCTAAAAATAAA
TGCAACGAAAACCACAAATCTATGTTTCACATTGAAAACGCTCATCAAAAAAACACCCCCACTCCAGCTA
GAAGGAGTTACACTAGACCAGCCGCTGCAAGCAACATATCTAGGTATAACACTGGATAAACGCCTCACCT
TTGGGCCGCATCTCAAAAATGCAGTAAAGAAATGTGGTCGCAGATCACAACAACTGAGATGGCTCATGAA
CAGAAGGAACACCCTCTCGCTGAGATGCAAAAGAGCTGTGTATGCGCACTGTATCCTACCGATCTGGTTA
TACGGGATCCAGATTTGGGGAATCGCAGCCAAATCAAATTACAAACGAATCCAGGTGATGCAAAATCGAG
CACTGCGACAAATAACCAACTGCCCCTGGTACGTACGGAACTCTACACTCCATAAGGACCTCAATCTCTA
CACAGTTGAGGAACAAATTGGGAGACACACAAGTCGATACAGTGACAGACTACAGAGTCATAGTAGCCTT
CTTGCAAGACGTCTTATCCCCGCTCGACCTCTGAGACGACTCAAAAGGCAAGGCTTTGCCAAGACATTTG
GACAG

>*Helena_D.erecta*_scaffold_4845_2218646_2221251
AATGCGTACATCATCGGAAATTGCTGAATGCATACGTTGTCAGGAATTCGCCCACACATCTAAATACTGT
CGAAGTCATCCAAATTGTGCTCGCTGTGGTGAAAATCACTTAACCCAGTTATGCGTGCGTTCCAATGGTC
AACAGCCTATCTGTATACACTATGGAGGAAATCACACGGCAAGTTACAACGGTTTCCAGTGGTAACAGGA
GTATCTTCGACGATCAATGGGCACTACAAAAAAGCAACCACCTAAGCAAGCCAGGTTAGATTAAAGTGCA
ACAAACCAGCAATAACCTCAGCAAAAACAGCATCATGTAACTAAGCAGCATTCAATAGCAAGCACTCCCA
AAGCCCATACCGGGAGTTTTTCCTACGCAGCTATTGCAAGAAATGGCAATACAACAGACAAGCCTCGTCT
ACATAACGTACATGCCCAATTAAAGGAATATAATATTAAACAGCAACGCCCGATTAACGTTCAGTCAATA
TTGGCACAGCAGCAGGAACAATTTATGAATTGGCAGCAACAGCAGCAATTCCTATCGTGACTACAGGAGC
AACAACAACAAAACAAGCTGAATAGCCAACGACTCGAACGGTTGGAAAAACTGGTTTTTGAAATGGCCAA
TATGCTGAAGCAATGGACTGGGGATGCATCGGCTCTCCAACTCCATTGTAACGCTTTACCATCGCAATGA
ACCCTCTGAAGATACTTATCTGGAATGTAAATAGTATTTCAGGTAAAGCCAGAGAAGTTGAGCTCTTCAC
ACACAACAACTGCGTTGACATTATTCTCCTAAACGAGATCAGACTCAACAGAGGGAACACCGTTAAGATA
CATGGATATAGCTTTTATAATATAAAGTATATTATATATATATATATTTATATATTATATACATATATAT
TCCCCGCATACAAACCATCAAGCCATAATCATGGAATGGGGGGAGCAGCAGTACTGGTGAGGAGCTCTCT
TCGTCATTTCCCGCAAAGAGTTATTGAAACGAGAACTATTCAGATGTCTTCAGTTAAGGTTTCCACCGGG
CTGGGAGATATGGAATTTAGCGCGATATACTGTCCACCAACAAATAGAATTGAAGAAAGGCACTTCAGTG
ACATACTTGTCTCTTGTGGCCAAAGGTATTTAGTTGGTAAGGACTGGAACATTAGCTATGATGTGACGGG
TGATACGTACAAATCACCCACGGGTCCAGAACTAGCAGAAGCCATTTCAGCCAGAGGTGCTTATATCCTT
GCAACAAGTTCACCAACTTGATACCCACATGTGCCCAGTCATAGACCTACCTGCATTGATTTTGCTGTGT
ACCATGGGATAAACGTAGACAGAACAAGTATTTGCGAAATTTGGGATCTAAACTCCGATCATGTAGCCCG
TGTTGCTACTCTACAAACAGAAGGTGTCTATGTTACGCCATGTTCTCGGTTAAAAAAAAACCGATTTTTT
TGTTTTCAGACATCTTGAAAACTCTTTTCAATTAAATACGGTTCTCAGCTCTAAGGAAGAGATCAAGAAC
CCAGTAGACATCCGAACGCAAAATATACATAGAGCCGCTTCTGTTTCCACGCCGTCTGAGCCTGAGACAT
GCCCCACATGTTATGGCATTGTACTAACAAGAGAGGCCTAACAAGAGAGAACTAATCAGAACTAAAAGAC
GTCTTCGAAGAAGAGCAATTCGAACTCGGGATCCATGGGACAGAATCTTGTGGAACCGAGCAGCAAAGCA
ACTCAAAACCATCTTAAGGGAACTCAGAAGTTATTTCTTTGAGGAAAAAAGTATCTTCCATGGACTACAC
TGTTGATGCAAACTATTCGCTGTGGAAGTGCACAAAAGCGCTTAAACATCAACCACTTAGATGGGTACCC
GTCGGCTACCACCTAGAGGATCCCTTCACTCCTTATGACTACGCCACGACAGACAAGAGAGAATCACCAG
TACCTACAAATGCCATTGCATTTGTCTTGGCCTATTAAGCCAATAACGATAGAAGAAATATCTGAAATAA
TCAAATTAATGCCGAAACACAAAGCTCCTGGGTCGTATCGGCCTATAAGCCTCTTACCGTCCCTTTCTAA
ATTATGGGAGAGACTTTTTGCCAATTGGATCAACGACATTATAAGACAAGGTAATATATTGCCGGATCCT
CAATGTGGATTTCGAAAGAGACACGGCACAATAGAACAGGTCCACAGACTGGTAAAACATATATTACAGG
CTTTTGACGACTGCGAGTCGTCTTTATAGACATGCAACAAGCCTTCGACAATGTATGGCATGTTTGATTA
TTATGCTAGATGAAGACCCTCCTACCTGCGCCCTACTTCTGTATTTTGAAGTCATATCTGAATTTAAAAT
CACGGTGAGAAATAACTACTCTTCTATATATCCAATGAGAACTGGAGTCCCACAGGGCAGTGTTCTCGAA
CCGTTACTGTATTCCTTGTACACTGCTGATATCGCTTGCCTGAATTTCGAACACATGGTAGCACCCAACA

AGGCTCTTATGGCAACCTATGCAGATGACATTGCAGTTGTGTACAACTCTAGGGACAGTAGAGAGACAGC
TACAAGGATACATTAA

>*Helena_D.ananassae*_scaffold_12984_435983_440038
TATGAACTCTAAAAAGGTGCATAGAGAACTGAAGTTTGATTCACCCTCAGTATCAATCAGCCAGGAAAGC
TCCAGCAATAGATTTGCTTTACTAGACATGGAAGTGGACCTAAACAGTATCACACATGCTGAGGAAAGAA
CAATCAATGTAGAGGGAAGCCCTACTAGATCTCCTATGGCTGTTCAAGAAAATATATCTGACGATAATGT
AGACACAGATGATTTAGGCAATAGATCCCAACAGAATGATTCTAATAGAAATTATTATCCTAAAGAAAAT
GCAAAACCACCGCAAATAGTTTTAAGCATTGCTAACCTAAATGATCTATTTGACCTCATTTCGGAGGTCA
CTAGCTTGGATAACGTAACAGTTAAAGTCAATCAAGGAGTTACGGCTAGAATATTTCCCAAGGACTCTAC
AACTTATAGAGCTATTGTCGACCATTTTGATAGAATGGAAATTGAATTCCATACATACCAAATGAAGGAA
GAGAAGCCTCACAGAATCGTAGTTAAAGGACTTCATCATAGCACACTAACTACTGAAATTGTTGCCAATT
TTAAAACATTTGGCTTCGATGCTCTTCAGGTGCATAACCCAAGATCAAGGTCGAACCATGATGTAAAATT
AAACATATTTTTTATTAATATTAAGCCTTGCGCTAGAATTAATGCTATATATGATATAGTAACATTGTGT
CGACACCCAAATTGTGCTCGATGTGGCGAAGACCATTTTACATGTGTTTACCATGTGTTCGACCCCAAGA
TCAACCGCCAATCTGCATGCACTGTGGAGGCAATCATTCGGCAAGCTACAAGGGTTGTCAGTCCTATCAA
AATTACCTCAGGCGATCTATGGGTTCTGCAAAGAAGCAAACAACGCGACAACAACCACAACAACGTCATC
AGCAACAACAGTCAACTCGACAACTAAATACTCCACATATTTCTGCAACCAATAGTGGAGGTTTATCTTA
TGCTTCAATAGTAAGAAATGGAAATGAACCAGCTCAACGCCGTCTACATGATCTCCAAGTCACAAGTACA
CAAATCGAAGGAAAATGTGCAACAACAATATGCGACTGATGTTCAGGCAATTTTAGAGCAACAGCAACAG
CTATTTATGAAATGGCAACAACAACTCCAAGAAAAGCAGCAGCAGCAATTCCTTATGTGGCTAAGGCAGC
AGCATCAAGAACAACAAATGCAAAACAAGCTGAACAGTCAACGACTTGAACGTCTTTAAAATATGGTTTT
TGAAATGGCAAACTCGATCAAGCAATGGACTGGAGATAAATCACTTCTTCAGCTTCCCAACAACGCTTCA
GCCTCACAATGAACCCACTCAAAATTCTTATTTGGAACGTCAATGGCATTTTAGGTAAAGCCAGAGAAAT
TGAATTCTTCGCGCACACCAACGAAGTTGATATCCTACTCCTAAGTGAATTAAGGCTCAATCGAGGAGAA
ATTGTCAAAATTTATGGATATTCCTTTTATCCAGCATACAAACCGGCAAGACACAATCACGGCACCGGAG
GAGCGGCGGTATTGGTAAGGAGCTCTCTTAGTCATTTTCCACAAAGTGTTATTGAAACACAAACCATTCA
AATGTCCTCTATCAAAGTTGACACAGGTTTGGGTATCATGGTAGTATGCGCAATATACTGTCCTCCAAGA
AACAGAATTGAAGAAAGGCATTTCACTGACATACTCGCTTCTTGTGGTCAGAGGTATCTGGTCGGTGGTG
ACTGGAATGCTAGACATTGGATGTGGGGCGACACTTACAATTCACCAAGAGGACGCGAGCTAGCTGAATC
CACTGTAGTAACAGGGGCTAAAATTCTTGCCACTGGTTCACCAACTAGATACCCTTATGTACCTGGCCAC
ACAGCTTCTTGCATAGACTTTGCATTATACCATGGGATACTGGATTCTCAAATAAGTATAGGCCAAAACT
GGGATTTGGACTCGGATCACATAGCTCTCGTTGTTAATCTGCAAACAAATGGTGTACAGATCAGACCAAG
CCCTCGTTTAATTACAAGCCGAACTGATCTCAATACTTTCAAACAACATCTTGAGACCTCCTTTCAACTA
AATTCTGTTCTGAACTCTAGAGAAGACATCGAGAATGCAGTTGACATTCTTACTGATAATATATATAGAG
CTGCTACTGTTTCAACGCCACCAGAACCTGTTCTTCGTCCTATAAGCTATGGCATTGTTCTTACAAGGGA
GGCGAGAGAGTTAATCAAGAGAAAAAGGCGCCTTCGAAGAAGAGCAATTCGTTCTCAAGATCCTTTGGAC
CGAAATAGATGGCACCAGGCTGAGAGGCAACTACGAACTGTTTTAGATGAACTCCGAAGCGATTGTTTCG
AGCAAAAGTTATCCTCTATGGACAATACCGTTGACGCAAACTACTCACTATGGAAGTGCACAAAATCGCT
TAAACGACAACCTTTTAGACAAGTTCCAGTCCGATGTCCTGATGGTGAACTGGCAAAAAATGAATTGGAG
CAGGCTAATGCTTTTGGCCAGCATCTAGAGGATCGCTTTACTCCCTTTAACTTTGCATCGGTAGAACAGA
CTAGAGAGACCTATCAGAGTTTGGAGACTCCATTGCAGATGTCACTGCCTATTAAGCCTATAAGAGTCGA
GGAAATATCTGATGTCATACAATCTTTGCCCAAAAACAAAGCTCCGGGTATTGATCGAGTTTGTCACGCT
ACGTTAAAGGCCTTGCCTACAAAAGCAATCCTTTTCATAGCATTAATTTTTCATGCAATAATTAGGATCC
AAGTGTTCCCAAAACAGTGGAAATTGGCTGCGATTATGATGATCCATAAGCCAGGGAAACCAGAGGTTGA
TCCTGAGTCATATCGTCCAATTAGTCTCCTACCCTGTTTGTCTAAACTGTGGGAGAGACTTATTGCAAAC
CGAGTTAAAAGGATTATGACTGAAAACAATATCTTGCCAGATCATCAATTTGGCTTTCGAGGAGGACACG
GCACCGTGGAACAGGTTCACAGACTGGTGCAACATATCTTGCAGGCCTTTGACGATCAGGAGTATTCCAA
CGCAGTTTTTATCGATATGCAGCAAGCTTTTGATAAGGTGTGGCATGATGGCTTATTGCTCAAAATTAAA
AATCTTTTACCTGCGCCGTACTATGGTCTGTTTAGATCATATTTAGAAGTACGAGAATTCAAGGTGAAAG
TAAAAGATTCATACTCGGACAACTTTTTGATGAGAGCAGGAGTTCCACAGGGAAGTGTGCTTGGACCGTT
GTTGTACTCACTGTATACGGCAGATATACCTATCCCGAGCAGCCAACATATGGTAGCCCCCTCTAAAGCA
CTTATTGCGACCTATGCAGATGACATTGCAGTCATTTACAACTACAGATGTCACAGAGAAGCTTCCAAAG
GATTACAGGAGTACCTATTCACTCTTGCAGCTTGGTGCAAAAGATGGAACCTGAAGATCAATCCGTAGAA
AACAACGAACGTCTGCTTTACACTGAAGAGGCTCATAATTAATACCCCTCAAATCCAACTTGAGGGAGTT
ACCCTAGAACAGCAAAACGAAGCAAAATATCTTGGTATTACTCTGGACAAGCGACTTACTTTCGGGCCAC
ATCTGAAGTCAACAACTAAAAAATGTAATATGAGGGTTCAGCAGCTGCGTTGGATGATAAGCAAAAGAAG
TACCATGCCGCTTAGGTGCAAAAGGGCAGTATATGTTCATTGCATCCTGCCAATGTGGCTTTATGGTGTA
CAAATTTGGGGGATCGCCGCCAAATCGAACTATAAGCGAATCCAGGTGTTGCAGAACCGAATGCTGAGAA
CCATAACAGGCTGTCCCTGGTACGTGCGTGGCTCCACTCTTCATAGAGACCTTAACATACATACTGTGGA
AGAACAGATAAATAGACACACAAGCCGTTACAGCGACAGACTTCTAAGACATCGCAGCCTACTGGCTAGA
AATTTACTTCCAGCTAGACCATTGAGGCGTCTTAAACGTCTAGGTTTTGCTAAGACACTTAATTAG

>*Helena_D.mojavensis*_scaffold_6540_6787901_6792402
ATGGACAAAAACCTTTCTGCTAACTCTCAGCTTAGAAATAAGCCGATTGATTATGCAAGAATGTGCCAAA
TAGCTGGGAAAACACCTTTTGAGCTGAGGGCTGAATTCAATAACCAACGCCTATCATGCACTGATTCTCA
GAATCTCAGCACCATGACAACAACAATCACATTTACTAGTGCTACTTGTAGCACAGTATATACCACCGCA
ACTGCTGCAATTAACAGCGCGATCCCTAGCGGAAGAATGGATAGTCAGGCAGCCGGCTTCCCACCTGACT
CTGTAAAACTTACTGGAGCTGCTACAAAACTGGCAGCAGCCCAGAAAATGCTTAAAACTAAAAGGAAGAT
ACCTCTATCTCCCAATAACAATAACAAAAGAGCAAACAGAGTTGAGGTCTCACACTCCGCTTCAACTAAT

ATGGCAAAATCCAGTAACAGCTTTGCTTTACTGGACATGGATATGGACGCAACCAGTGAAGGCGAGGATG
ATGTGAATCCCACTGCTTCTATTGTTGATCATAGCAACACTGCTGATGCAATTGATACAGTCAACAGCCA
TCAGAATAAAGAACCCAGCAACAACCAACAGACTCTCGGGTCATCTAAGCCACCGCAAATCGTTGTGAGT
ATCACTGATCTTAATGATCTATTTGAAATCATTAGTGAAGTCACCAGTATTGATAACGTATCAGTCAAGA
TCAACCAAGGAGTGACGGCTAGAATATTTCCCAAAGATAGCGACACTTACAGAGCTATCGTAAGCCATTT
TGATGCGATAGGTATTGAGTTCCATACGTATCAGATGAAGGAAGAAAAACCTTACAGAATTGTAGTAAGA
GGGCTACACCATAGTACCTTGAACAATGAAATAATTGCCAATTTTAAAAAATATGGCTTCGATGCTCTGC
AAATTCACAACCCAAGGTCACGGTCAAACAGGGATGCAAAATTAAATATATTTTTTGTAAACATAAAACC
TTGTAAAAACATTAACGAAGTTTACAATATAAAAACACTTTGCCGCCAAATGGTGAGAGTTGAAAGGATG
CGTAAAACTTCTGAAATCGTCATATGTACCCGATGCCAAGAATATGGCCATACAGCCAAATACTGCCGTC
GCCATCCTAACTGTGCACGCTGTGGCGAGGACCATCCCACCAATCTTTGCGCACGTTCACAAGATGCACC
GCCTACCTGCCTTCACTGTGGAGGTAACCACATGGCAAGCTACAAAGGCTGCCCGTGGTACCAGGAGTTC
TATCGACGTTCACTGGGCTCCTCTACGATCAAGAAGAATCAAGCAAGACCACAACAGCCACAACGGAAGC
ACCAGCATCAACCAACACGTCCACAACCACAACCACAGCAGCTATCAAATACTTCTGGAGTTGGAGCAGG
TGGAATATCCTATGCCGCGGTAGCAAGAAATGGAAGCGCATCTGCTCAAAGCCGCATACATAATATTCAA
GCGCAAGCCCAGTTAGCTAAACCTATAGGACATATCGCACAGCAGCAGCATCAAGTTGATGTCCAATCTT
TACTGGAACAACAACAACAACAATTTTTGAAATGGCAAAAAGACTTGCAAGTGCAGCAACAGCAGCAATT
TCTTTCATGGCTTCAAGCACAACAACGCGAACAGCAACAACAAAACAAGAGAAACAGTGAACGACTAGAA
CGTCTAGAAAAAATGGTCCACGAAATGGCCAGCATGCTCAAGCAATGGACTGGGGATTCATCGACTCACC
AGCTTCTTAACAACGCCTCAGCCTCACAATGAACCCACTCAAGATTCTCATCTGGAATGTCAATGGCATT
GCAGGAAAAGCCAGAGATGTGGAGCTATTCGCGCACAACAACAACATCGACATTCTACTATTAAATGAAA
TCAGACTGAATCGAGGAGATACAGTCAAAATATATGGCTACACCTTCTATCCAGCTTACAAGCCATCAAG
CCACAACCATGGTATGGGAGGAGCGGCTATATTCGTTAGAAGTTCACTGCGCCACTTCCCACAAAGGGTT
ATTGAAACTCAAAATATACAAATGTCTGCAATCAAAGTTGCCACTGGATTGGGAGACATCGAATTTTGCG
CCATATACTGCCCACCAAGGAACAGAATAGAAGAAAGACAATTCAGCGACATACTCGCTTCTTGCGGCCA
AAGGTATCTTATTGGTGGTGACTGGAATGCACGACACTGGCTGTGGGGAGATTCATACAATTCACCCAGG
GGAAGAGAACTAGCAGAAGCCATCACGGCCAGAGGAGCTAATATTCTTGCAACAGGTTCTCCTACTAGGT
ACCCATATATATCCAGTCATACTCCCTCTTGCATTGATTTTGCATTATACCATGGGATACAGCATTATCA
AGCAAATATTTACCAAAGCTGGGACTTAGACTCTGACCATTTAGCCCTTATTGCCGAGTTACACATCGAT
GGTCATTATATCAGGCCAAGCCCAAGGCTAATAACCAGCCGTACTGACATCGAGGCTTTCAGACAACAGC
TAGATGACTCCATTCATTTGAATTTGTGTCTGAACTCCGGCGAAGACATCGAGAATGCGGTTGATAATCT
CTCGGAGAACATATATAGAGCTGCTGCTGCTACAACGCCGCTTAATCCTGCGCCTCGTCCCAATAATTAT
GGCATTGTTCTAACGAGAGAAGCCAAAGAACTCATCAGAACAAAAAGGCGCCTTCGTAGAAGAGCAATTC
GATCCCAAGATCCTTGGGACCGCCTTTTGTGGAACCGGGTAGCAAGACAGCTACGTAACCTTTTAAGGGA
ACTCAGAAGCGATTTCTTCGAGCAAAAACTGGCTTCCATGGACTACACTATTGATGCAAACTATTCGTTA
TGGAAATGCACAAAATCACTCAAACGACAACCTTTTAGACAGGTACCTGTCCGATGCCCTAACGGCGAAG
TTGCTAAATGCGAATTGGAACAGGCTAACGCATTCGGATGTCATCTAGAAGAACGTTTTACTCCATTTAG
TTATGCTACGACGGAGCAGACCATGGAGATACAGCTATATTTGCAGACCCCCTTGCAGATGTCTCTGCCT
ATTGAGCCTATAAGGATTGAAGAAATTACCGAAGCGATCCAAATATTGCCGAAAAATAAAGCTCCCGGAA
TAGACAAGATTTGCAACGCAACGCTGAAGGCCTTACCTACAAGAGCGCTACTTTACATTGCACTAATCTT
CAATGCCATCATCAGGCTCCAAGTGTTCCCAAGGCAATGGAAATTAGCTGCTATCTTGATGATCCACAAG
CCTGGCAAGCCGGAAGAGGATCCAGAGTCATATCGGCCTATAAGTCTACTGCCCTCCTTGTCTAAACTAT
GGGAGAGGACCATTGCCAACCGCATTAACGCAATATTTAGACAATCCAATATCCTACCGGATCACCAATT
TGGATTCCGGGAGGGACACAGCACAGTGGAACAGGTCCATAGACTGGTGAAACACATCTTGCAGGCTTTT
GATGACTCTGAATACTCCAATGCTGTCTTTATCGACATGCAGCAAGCGTTTGATAAGGTGTGGCACGATG
GATTACTGTACAAAATAAAAAACCTTCTACCTGCTCCGTACTATGGCCTCTTAAGATCTTATCTAGAAGA
CCGTGTGTTCCAAGTCAAGGTAAAAGATACACTCTCGTCCACATACCCTATGAGAGCAGGAGTGCCGCAG
GGCAGTGTTCTTGGTCCGCTGCTGTTCTCCTTGTACACCTCTGATATACCTAGCCCGTCCTCTCAACATA
TGGATGCTCCCTCGAAAGCTGTCATTGCCACATATGCTGATGATATTGCCATCATCTATAACTCAAAAAA
CGTGGTTGAAGCAAGTACAGGACTACAGAGATATCTGGATACTCTCGCTGATTGGTGCAAGCGGTGGAAT
CTCAAAGTTAACCCGCTAAAAACATTGAACCCTTGCTTCACTCTAAAAAGGCTGGCAATGCATACCCCCC
CAATCCAGATGTCGGGAGTAACCCTACAGCAACCTGCTCAGGTGAAATATCTTGGCATCACACTGGACAA
GCGCCTCACCTTCGGTCCACACCTCAAAGCTACGGTGAAAAAATGTCGTCACAGACTGCAACAATTAAGA
TGGCTCACCAATAGAAAGAGCACCTTACCGCTGAGATGCAAAAGAGCTGTCTATGTGCACTGCATCCTAC
CAATATGGCTCTATGGAGTGCAAATCTGGGGGATCGCTGCAAAATCGAATTATAAAAGAGTGCAGGTTTT
GCAGAACAGAGTGTTAAGGCAGATAACAAACTGTCCCTGGTACATACGCGGCTCAACACTGCATAGAGAC
CTCAAGCTGTCTACTGTCGAAGAACAAATAAATAAACACACAAGCAGATACTCAGACAGACTTGTACGAC
ATCAAAGCCTACTCGCAAGAGGTCTAACACCTGCCAGACCCCTAAGACGACTTAAACGGCAGGGCTTTGC
CAAAACAATGGGTATTCAATAA

>*Helena_D.virilis*_scaffold_13050_111322_111882
TTCCAAAGCAATTGGAAACTAGCAGCGATTATGATGATTCTCAAGCCAGGCAAACCAGAAGCTGATCCTG
AGTCGTATCGGCCAATAAGTCTCTTACCCTCCCTGTCTAAACTATGGGAGAGGACTATTGCCAATCGGAT
TACTGCCATTACAAGCCAATGCAATATCTTGCCGGATCACCGATTCGGATTTCGAGAGGGACACGTCACT
GTGGAACAGGTGCACAGACTGCTGAACCATATCATGCAGGCTTTTGACTACCTAGAGTTCTCAAACGCTG
TCTTCATTGACATGCAGCAAGCCTTCGATAAGGTGTGGCACGATGGATTACTGTGCAAAATAAAAAACCT
TTAACCTGCACCGTACTATGGCCCTCTAAGGTCATATTTGGAAGTACGAGAGTTCAAGGTCAAGGTAAGG
GACACATACTCGTCCACCTATACTATGAGAGCAGGAGTGCCACAGGGTAGTGTTCTTGATCCGCTGCTGT
ACTCGTTGTATACCTCAGATATACCAGCCCTACCTCTCAACATATGGACGATCCCTCTAAGGCTATTATT
G
