# Supplementary material for: The evolutionary dynamics of the Helena retrotransposon revealed by sequenced Drosophila genomes
Source: BMC Evol Biol. 2009 Jul 22;9:174. doi: 10.1186/1471-2148-9-174 (PMC3087515; doi:10.1186/1471-2148-9-174)
Supplement: Additional file 2 — Helena copies in the Drosophila sechellia sequenced genome. The data provided is a list of D. sechellia copies. [file 1471-2148-9-174-S2.doc]

**Additional File 2.** *Helena* copies in the *Drosophila sechellia* sequenced genome.

| **Contig** | **strand** | **start** | **stop** | **length (bp)** | **% identity with the reference *Helena* insertion** |
| --- | --- | --- | --- | --- | --- |
| super_0 | + | 12171356 | 12171444 | 89 | 91.0 |
| super_1^*^ | + | 35325 | 36999 | 1675 | 93.7 |
| super_1 | + | 39172 | 39264 | 93 | 96.8 |
| super_2 | - | 797880 | 797990 | 111 | 96.4 |
| super_5 | - | 4228108 | 4228622 | 515 | 97.9 |
| super_6 | - | 3812199 | 3812282 | 84 | 91.7 |
| super_8 | - | 1891290 | 1891380 | 91 | 93.4 |
| super_8 | - | 1367531 | 1367620 | 90 | 92.2 |
| super_11 | - | 2878347 | 2878572 | 226 | 97.3 |
| super_11 | - | 2618028 | 2618117 | 90 | 91.1 |
| super_16 | + | 322709 | 322925 | 217 | 96.8 |
| super_18^$^ | - | 166502 | 169486 | 2985 | 98.2 |
| super_21 | - | 559984 | 560071 | 88 | 89.8 |
| super_25 | + | 487507 | 487601 | 95 | 93.3 |
| super_26^$^ | + | 76452 | 76608 | 157 | 89.2 |
| super_27 | + | 794206 | 794421 | 216 | 97.2 |
| super_30 | + | 663871 | 664403 | 533 | 96.6 |
| super_41 | - | 252 | 478 | 227 | 96.9 |
| super_42^$^ | + | 237131 | 239653 | 2523 | 88.4 |
| super_42^$^ | + | 241344 | 242048 | 705 | 89.8 |
| super_43^$^ | - | 216904 | 217089 | 186 | 91.9 |
| super_43 | + | 276915 | 280722 | 3808 | 98.1 |
| super_46 | - | 303478 | 304119 | 642 | 97.7 |
| super_46 | - | 285197 | 286807 | 1611 | 96.1 |
| super_46 | - | 38589 | 39337 | 749 | 95.5 |
| super_46 | - | 281276 | 281682 | 407 | 93.6 |
| super_48 | - | 68931 | 69072 | 142 | 90.1 |
| super_49^$^ | - | 2528 | 5264 | 2737 | 97.8 |
| super_49 | + | 7263 | 7465 | 203 | 97.0 |
| super_51 | - | 154509 | 154861 | 353 | 98.6 |
| super_54 | + | 159232 | 159315 | 84 | 91.7 |
| super_57^$^ | + | 191582 | 192774 | 1193 | 98.3 |
| super_57^#^ | + | 193510 | 194264 | 755 | 95.8 |
| super_58 | + | 238 | 410 | 173 | 96.5 |
| super_60 | + | 98669 | 99095 | 427 | 93.0 |
| super_63^$^ | - | 152265 | 153066 | 802 | 95.6 |
| super_63 | + | 115441 | 115566 | 126 | 86.5 |
| super_65 | - | 80845 | 80982 | 138 | 86.2 |
| super_66 | + | 126221 | 126334 | 114 | 95.6 |
| super_70 | - | 116473 | 116611 | 139 | 88.5 |
| super_74^$^ | + | 99148 | 101182 | 2035 | 98.1 |
| super_79^$^ | + | 31706 | 31893 | 188 | 91.0 |
| super_80 | - | 115484 | 115620 | 137 | 85.4 |
| super_80^$^ | - | 121280 | 125760 | 4481 | 96.6 |
| super_82 | - | 70476 | 70566 | 91 | 87.9 |
| super_82 | + | 74589 | 74690 | 102 | 86.3 |
| super_88 | - | 27830 | 27913 | 84 | 94.0 |
| super_88 | + | 46967 | 47050 | 84 | 94.0 |
| super_93 | - | 4 | 3129 | 3126 | 99.9 |
| super_95 | + | 89222 | 89479 | 258 | 95.5 |
| super_99 | - | 95182 | 96837 | 1656 | 95.0 |
| super_99^$^ | - | 96945 | 98640 | 1696 | 95.3 |
| super_99 | - | 98725 | 99126 | 402 | 95.5 |
| super_99^$^ | - | 93770 | 94275 | 506 | 95.2 |
| super_99^$^ | - | 94295 | 94954 | 660 | 96.2 |
| super_102 | + | 56801 | 58602 | 1802 | 97.9 |
| super_102  Additional File 1. Cont. | - | 102193 | 102331 | 139 | 87.1 |
| super_104^$^ | + | 51420 | 54479 | 3060 | 89.0 |
| super_106^$^ | - | 85608 | 85801 | 194 | 86.5 |
| super_106^$^ | - | 89297 | 89490 | 194 | 86.5 |
| super_107^$^ | + | 6494 | 7791 | 1298 | 89.4 |
| **super_107^§^** | **+** | **28043** | **32931** | **4888** | **-** |
| super_111^$^ | + | 84813 | 86759 | 1947 | 88.6 |
| super_117 | + | 45468 | 45791 | 324 | 97.8 |
| super_149^*^ | + | 22228 | 24040 | 1813 | 98.3 |
| super_176 | + | 79638 | 79730 | 93 | 82.8 |
| super_189 | + | 26453 | 26679 | 227 | 96.9 |
| super_202^$^ | + | 12370 | 12758 | 389 | 92.0 |
| super_206 | - | 3416 | 3623 | 208 | 97.1 |
| super_212 | - | 805 | 891 | 87 | 91.9 |
| super_240 | + | 5789 | 5927 | 139 | 83.5 |
| super_267 | - | 7806 | 8021 | 216 | 96.8 |
| super_267^*^ | + | 8208 | 12640 | 4433 | 96.3 |
| super_269^$^ | - | 5847 | 6772 | 926 | 97.5 |
| super_274 | - | 9270 | 9675 | 406 | 95.1 |
| super_274^$^ | - | 7075 | 8868 | 1794 | 97.4 |
| super_278 | - | 11271 | 11979 | 709 | 99.3 |
| super_292^$^ | - | 11685 | 12980 | 1296 | 95.1 |
| super_327 | + | 670 | 896 | 227 | 96.9 |
| super_338 | - | 39692 | 39917 | 226 | 97.3 |
| super_360^*^ | + | 1444 | 5407 | 3964 | 98.0 |
| super_367 | - | 2213 | 2351 | 139 | 88.5 |
| super_369 | + | 12506 | 12614 | 109 | 89.9 |
| super_400 | - | 8898 | 8984 | 87 | 92.0 |
| super_440 | + | 36226 | 36437 | 212 | 89.6 |
| super_459^$^ | - | 4759 | 6506 | 1748 | 99.0 |
| super_534 | - | 432 | 1469 | 1038 | 96.7 |
| super_552^$^ | - | 10218 | 10922 | 705 | 89.8 |
| super_552^$^ | - | 2634 | 2857 | 224 | 90.6 |
| super_632 | - | 3122 | 4386 | 1265 | 95.2 |
| super_703 | + | 5243 | 5381 | 139 | 86.3 |
| super_708^$^ | + | 1215 | 1608 | 394 | 95.7 |
| super_715 | + | 5516 | 5742 | 227 | 97.4 |
| super_811^#^ | + | 3530 | 4688 | 1159 | 96.2 |
| super_855 | - | 5252 | 5421 | 170 | 97.6 |
| super_950 | - | 3157 | 4289 | 1133 | 96.6 |
| super_991 | - | 1485 | 1705 | 221 | 89.1 |
| super_1104 | - | 3527 | 3753 | 227 | 97.4 |
| super_1012 | - | 5941 | 6047 | 107 | 72.9 |
| super_1016 | + | 4699 | 5202 | 504 | 97.2 |
| super_1189 | + | 5007 | 5218 | 212 | 89.2 |
| super_1215 | + | 427 | 642 | 216 | 97.2 |
| super_1223 | - | 4514 | 4602 | 89 | 84.3 |
| super_1280 | - | 4772 | 4929 | 158 | 93.0 |
| super_1432^$^ | - | 3209 | 5699 | 2491 | 97.7 |
| super_1432^$^ | - | 1652 | 1874 | 223 | 93.3 |
| super_1432^$^ | - | 1 | 1414 | 1414 | 98.3 |
| super_1490^*^ | + | 51 | 3996 | 3946 | 98.2 |
| super_1555 | - | 4101 | 4327 | 227 | 98.2 |
| super_1637 | + | 2144 | 2269 | 126 | 88.1 |
| super_1837 | - | 3860 | 4099 | 240 | 95.8 |
| super_1837 | - | 3669 | 3837 | 169 | 98.8 |
| super_1837^$^ | - | 2168 | 3641 | 1474 | 95.3 |
| super_1989 | - | 3626 | 3761 | 136 | 87.5 |
| super_2344^$^ | - | 71 | 258 | 188 | 91.5 |
| super_2344^$^ | - | 4250 | 4437 | 188 | 91.0 |
| super_2404  Additional File 1. Cont. | + | 1897 | 2117 | 221 | 89.1 |
| super_2461^$^ | - | 21 | 2730 | 2710 | 96.2 |
| super_2470 | + | 3784 | 4008 | 225 | 97.3 |
| super_2525 | - | 999 | 1136 | 138 | 86.2 |
| super_2610^$^ | + | 2913 | 3141 | 229 | 95.6 |
| super_2663^*^ | + | 2 | 3091 | 3090 | 97.5 |
| super_2853^*^ | + | 768 | 2942 | 2175 | 98.4 |
| super_3054^$^ | + | 1 | 721 | 721 | 92.4 |
| super_3054 | + | 1033 | 1164 | 132 | 99.2 |
| super_3062^$^ | + | 1 | 2805 | 2805 | 96.8 |
| super_3090^$^ | - | 458 | 1190 | 733 | 94.5 |
| super_3257^$^ | - | 123 | 2604 | 2482 | 98.4 |
| super_3285 | - | 2212 | 2674 | 463 | 97.4 |
| super_3398^$^ | + | 1 | 1490 | 1490 | 97.6 |
| super_3398 | + | 2447 | 2620 | 174 | 97.7 |
| super_3400 | + | 391 | 517 | 127 | 98.4 |
| super_3678 | - | 2 | 490 | 489 | 92.0 |
| super_4238 | + | 1 | 629 | 629 | 90.8 |
| super_4701^*^ | - | 1 | 2193 | 2193 | 97.6 |
| super_4766^#^ | - | 1000 | 2180 | 1181 | 95.6 |
| super_4830 | + | 1710 | 2165 | 456 | 99.3 |
| super_4968 | - | 1 | 2134 | 2134 | 97.5 |
| super_5046^$^ | + | 4 | 2110 | 2107 | 97.6 |
| super_5110 | + | 1114 | 1252 | 139 | 85.6 |
| super_5246 | + | 1623 | 2066 | 444 | 97.1 |
| super_5283 | - | 1443 | 2003 | 561 | 97.1 |
| super_5290 | + | 1494 | 2057 | 564 | 96.8 |
| super_5355 | - | 1816 | 2031 | 216 | 97.2 |
| super_5443 | - | 1022 | 1246 | 225 | 97.3 |
| super_5736 | - | 244 | 463 | 220 | 88.6 |
| super_6340 | - | 1751 | 1855 | 105 | 87.6 |
| super_6884 | - | 1230 | 1753 | 524 | 95.2 |
| super_6939^$^ | - | 9 | 196 | 188 | 91.5 |
| super_7024^*^ | + | 132 | 1761 | 1630 | 95.2 |
| super_7397^#^ | - | 1 | 1025 | 1025 | 98.9 |
| super_7397 | - | 1027 | 1148 | 122 | 93.4 |
| super_7468 | + | 302 | 1109 | 808 | 96.9 |
| super_7484 | + | 965 | 1190 | 226 | 97.8 |
| super_7535 | - | 95 | 321 | 227 | 97.4 |
| super_7747^$^ | + | 68 | 1063 | 996 | 98.9 |
| super_7747 | + | 1301 | 1665 | 365 | 97.6 |
| super_7886^$^ | + | 883 | 1630 | 748 | 97.9 |
| super_8445^$^ | - | 1180 | 1589 | 410 | 97.8 |
| super_8462^#^ | - | 1 | 611 | 611 | 96.3 |
| super_8650 | + | 1036 | 1171 | 136 | 95.6 |
| super_9088 | - | 764 | 1451 | 688 | 93.0 |
| super_9587^$^ | + | 3 | 169 | 167 | 93.9 |
| super_9752 | - | 628 | 1081 | 454 | 96.5 |
| super_9783 | + | 277 | 490 | 214 | 90.2 |
| super_10326 | - | 1009 | 1208 | 200 | 88.0 |
| super_10688 | - | 1 | 539 | 539 | 98.1 |
| super_11109 | - | 121 | 691 | 571 | 97.5 |
| super_11246 | - | 824 | 962 | 139 | 98.3 |
| super_11299^$^ | - | 19 | 515 | 497 | 87.1 |
| super_12123 | - | 1 | 120 | 120 | 98.0 |
| super_12201 | + | 1 | 1129 | 1129 | 95.8 |
| super_12367^$^ | + | 1 | 1143 | 1143 | 94.4 |
| super_12579 | + | 98 | 184 | 87 | 92.0 |
| super_12620^$^ | - | 1 | 389 | 389 | 92.5 |
| super_13215^$^ | - | 650 | 843 | 194 | 86.5 |
| super_13541  Additional File 1. Cont. | - | 559 | 784 | 226 | 97.3 |
| super_13682 | - | 32 | 996 | 965 | 95.5 |
| super_13703# | - | 335 | 522 | 188 | 91.5 |
| super_13846 | + | 224 | 985 | 762 | 98.5 |
| super_14167 | - | 763 | 903 | 141 | 95.7 |

^§^ the reference *Helena* copy

^*^ sequences with internal deletions and insertions

^$^ sequences with internal deletions

^#^ sequences with insertions
