# Supplementary material for: The evolutionary dynamics of the Helena retrotransposon revealed by sequenced Drosophila genomes
Source: BMC Evol Biol. 2009 Jul 22;9:174. doi: 10.1186/1471-2148-9-174 (PMC3087515; doi:10.1186/1471-2148-9-174)
Supplement: Additional file 3 — Helena copies in the Drosophila erecta sequenced genome. The data provided is a list of D. erecta copies. [file 1471-2148-9-174-S3.doc]

**Additional File 3.** *Helena* copies in the *Drosophila erecta* sequenced genome.

| **Contig** | **strand** | **start** | **stop** | **length (bp)** | **% identity with the reference *Helena* insertion** |
| --- | --- | --- | --- | --- | --- |
| **scaffold_4845^§^** | **+** | **2218646** | **2221251** | **2606** | **-** |
| scaffold_4845 ^*^ | - | 258342 | 260179 | 1838 | 80.0 |
| scaffold_4859 | + | 368 | 549 | 182 | 91.2 |
| scaffold_4859 | + | 780 | 1003 | 224 | 90.4 |
| scaffold_4929 | - | 24968900 | 24969728 | 829 | 85.4 |
| scaffold_4929 ^$^ | + | 25790686 | 25790993 | 308 | 90.6 |
| scaffold_4929 ^*^ | + | 25791025 | 25791401 | 377 | 92.3 |

^§^ the reference *Helena* copy

* sequences with internal deletions and insertions

^$^ sequences with internal deletions
