# Supplementary material for: The evolutionary dynamics of the Helena retrotransposon revealed by sequenced Drosophila genomes
Source: BMC Evol Biol. 2009 Jul 22;9:174. doi: 10.1186/1471-2148-9-174 (PMC3087515; doi:10.1186/1471-2148-9-174)
Supplement: Additional file 4 — Helena copies in the Drosophila ananassae sequenced genome. The data provided is a list of D. ananassae copies. [file 1471-2148-9-174-S4.doc]

**Additional File 4.** *Helena* copies in the *Drosophila ananassae* sequenced genome.

| **Contig** | **strand** | **start** | **stop** | **length (bp)** | **% identity with the reference *Helena* insertion** |
| --- | --- | --- | --- | --- | --- |
| scaffold_1935 | - | 641 | 776 | 136 | 89.0 |
| scaffold_3881^$^ | - | 1173 | 1347 | 175 | 85.7 |
| scaffold_8370^$^ | - | 4842 | 5181 | 340 | 90.9 |
| scaffold_10513 | + | 102801 | 103080 | 280 | 85.7 |
| scaffold_12413^$^ | + | 6702 | 6892 | 191 | 81.7 |
| scaffold_12911 | - | 779854 | 779937 | 84 | 85.7 |
| scaffold_12911^$^ | + | 780921 | 781040 | 120 | 85.8 |
| scaffold_12911^$^ | + | 779752 | 780287 | 536 | 87.8 |
| scaffold_12911 | + | 2045898 | 2046217 | 320 | 88.8 |
| scaffold_12911 | - | 1259036 | 1259190 | 155 | 100 |
| scaffold_12911 | - | 2043480 | 2043659 | 180 | 92.8 |
| scaffold_12911 | - | 2046249 | 2046356 | 108 | 90.7 |
| scaffold_12911 | - | 2045786 | 2045881 | 96 | 91.7 |
| scaffold_12911^$^ | - | 1252143 | 1252290 | 148 | 95.3 |
| scaffold_12911 | - | 2059723 | 2059805 | 83 | 84.3 |
| scaffold_12913^$^ | - | 194646 | 195458 | 813 | 95.4 |
| scaffold_12916 | - | 568070 | 568418 | 349 | 100 |
| scaffold_12916 | - | 16179002 | 16179418 | 417 | 81.1 |
| **scaffold_12984^§^** | **+** | **435983** | **440038** | **4056** | **-** |
| scaffold_12984 | + | 462586 | 463360 | 775 | 98.2 |
| scaffold_12984^*^ | + | 464018 | 467362 | 3345 | 98.1 |
| scaffold_12984^$^ | + | 422080 | 423824 | 1745 | 81.1 |
| scaffold_12943 | - | 351639 | 352991 | 1353 | 98.3 |
| scaffold_12943^*^ | - | 347978 | 350733 | 2756 | 98.9 |
| scaffold_13010 | + | 43733 | 44105 | 373 | 88.8 |
| scaffold_13010 | - | 413142 | 413344 | 203 | 96.0 |
| scaffold_13010^$^ | + | 413365 | 415265 | 1901 | 93.8 |
| scaffold_13010 | + | 412340 | 412545 | 206 | 91.3 |
| scaffold_13010^$^ | + | 105767 | 106086 | 320 | 90.6 |
| scaffold_13010^$^ | - | 38210 | 38400 | 191 | 50.8 |
| scaffold_13010^*^ | + | 44501 | 45455 | 955 | 75.7 |
| scaffold_13010^$^ | + | 123235 | 123445 | 211 | 86.3 |
| scaffold_13020 | + | 40841 | 42035 | 1195 | 94.9 |
| scaffold_13043 | + | 216476 | 216624 | 149 | 92.6 |
| scaffold_13043^*^ |  | 217334 | 220145 | 2812 | 85.4 |
| scaffold_13045^$^ | - | 583710 | 585024 | 1315 | 92.1 |
| scaffold_13045^$^ | - | 585425 | 587144 | 1720 | 91.2 |
| scaffold_13082 | - | 229612 | 229799 | 188 | 87.8 |
| scaffold_13082^*^ | - | 219285 | 220358 | 1074 | 87.6 |
| scaffold_13099^$^ | + | 306243 | 307353 | 1111 | 88.5 |

^§^ the reference *Helena* insertion

* sequences with internal deletions and insertions

^$^ sequences with internal deletions
