# Supplementary material for: The evolutionary dynamics of the Helena retrotransposon revealed by sequenced Drosophila genomes
Source: BMC Evol Biol. 2009 Jul 22;9:174. doi: 10.1186/1471-2148-9-174 (PMC3087515; doi:10.1186/1471-2148-9-174)
Supplement: Additional file 5 — Helena copies in the Drosophila yakuba sequenced genome. The data provided is a list of D. yakuba copies. [file 1471-2148-9-174-S5.doc]

**Additional File 5.** *Helena* copies in the *Drosophila yakuba* sequenced genome.

| **Contig** | **strand** | **start** | **stop** | **length (bp)** | **% identity with the reference *Helena* insertion** |
| --- | --- | --- | --- | --- | --- |
| scaffold_0.33 | - | 442824 | 442903 | 80 | 94.5 |
| scaffold_13.4 | + | 10130 | 10344 | 215 | 99.6 |
| scaffold_13.13 | - | 73091 | 73604 | 514 | 95.5 |
| scaffold_17.15^*^ | - | 53935 | 55201 | 1267 | 99.5 |
| scaffold_22.43 | - | 8361 | 8607 | 247 | 95.0 |
| scaffold_24.7^$^ | + | 51082 | 52547 | 1466 | 99.5 |
| scaffold_32.8 | + | 7911 | 8422 | 512 | 96.5 |
| scaffold_32.10 | + | 18186 | 18693 | 508 | 97.0 |
| scaffold_40.3 | + | 2105 | 2225 | 121 | 95.3 |
| scaffold_59.5^$^ | - | 3728 | 5084 | 1357 | 95.2 |
| scaffold_59.4^$^ | - | 6222 | 7094 | 873 | 92.2 |
| scaffold_77.5 | + | 58491 | 58962 | 472 | 91.8 |
| scaffold_85.13 | + | 15696 | 15810 | 115 | 96.4 |
| scaffold_113.6^$^ | - | 748 | 2390 | 1643 | 96.1 |
| scaffold_131.4 | - | 10410 | 11468 | 1059 | 99.0 |
| scaffold_149.6 | - | 15196 | 15501 | 306 | 95.8 |
| scaffold_149.6 | - | 16588 | 16685 | 98 | 99.6 |
| scaffold_153.3 | - | 11885 | 13165 | 1281 | 86.8 |
| **scaffold_197.8^§^** | **+** | **2845** | **4599** | **1755** | **-** |
| scaffold_278.1 | - | 5171 | 5476 | 306 | 96.3 |
| scaffold_278.2 | - | 3334 | 3639 | 306 | 83.4 |
| scaffold_278.3 | - | 1119 | 1216 | 98 | 96.5 |
| scaffold_2491.1 | - | 626 | 1878 | 1253 | 99.0 |
| scaffold_3304.1 | - | 1210 | 2126 | 917 | 91.7 |
| scaffold_7058.1 | + | 1 | 1019 | 1019 | 100 |

^§^ the reference *Helena* copy

* sequences with internal deletions and insertions

^$^ sequences with internal deletions
