# Supplementary material for: The evolutionary dynamics of the Helena retrotransposon revealed by sequenced Drosophila genomes
Source: BMC Evol Biol. 2009 Jul 22;9:174. doi: 10.1186/1471-2148-9-174 (PMC3087515; doi:10.1186/1471-2148-9-174)
Supplement: Additional file 6 — Helena copies in the Drosophila virilis sequenced genome. The data provided is a list of D. virilis copies. [file 1471-2148-9-174-S6.doc]

**Additional File 6.** *Helena* copies in the *Drosophila virilis* sequenced genome

| **Contig** | **strand** | **start** | **stop** | **length (bp)** | **% identity with the reference *Helena* insertion** |
| --- | --- | --- | --- | --- | --- |
| scaffold_12723 | - | 964607 | 964760 | 154 | 94.0 |
| scaffold_12734 | - | 311221 | 311765 | 545 | 93.6 |
| scaffold_12734 | - | 284098 | 284613 | 516 | 95.0 |
| scaffold_12734 | + | 298819 | 298978 | 160 | 95.0 |
| scaffold_12734 | + | 303987 | 304146 | 160 | 96.1 |
| scaffold_12958^$^ | - | 1866873 | 1867328 | 456 | 93.8 |
| scaffold_12958^$^ | + | 127677 | 128128 | 452 | 88.9 |
| scaffold_12958^$^ | - | 334101 | 334544 | 444 | 86.9 |
| scaffold_12958^$^ | + | 268971 | 269384 | 414 | 86.5 |
| scaffold_12967 | - | 99732 | 100300 | 569 | 85.7 |
| scaffold_13042 | + | 4906827 | 4906907 | 81 | 94.1 |
| **scaffold_13050^§^** | **+** | **111322** | **111882** | **561** | **-** |
| scaffold_13324^$^ | - | 2315058 | 2315544 | 487 | 90.1 |

^§^ the reference *Helena* copy

^$^ sequences with internal deletions
