# Supplementary material for: The evolutionary dynamics of the Helena retrotransposon revealed by sequenced Drosophila genomes
Source: BMC Evol Biol. 2009 Jul 22;9:174. doi: 10.1186/1471-2148-9-174 (PMC3087515; doi:10.1186/1471-2148-9-174)
Supplement: Additional file 7 — Helena copies in the Drosophila mojavensis sequenced genome. The data provided is a list of D. mojavensis. [file 1471-2148-9-174-S7.doc]

**Additional File 7.** *Helena* copies in the *Drosophila mojavensis* sequenced genome.

| **Contig** | **strand** | **start** | **stop** | **length (bp)** | **% identity with the reference *Helena* insertion** |
| --- | --- | --- | --- | --- | --- |
| scaffold_885 | + | 2 | 1019 | 1018 | 99.4 |
| scaffold_1657 | + | 2335 | 2586 | 252 | 99.5 |
| scaffold_1657 | + | 2791 | 2977 | 188 | 80.0 |
| scaffold_1873 | - | 352 | 986 | 635 | 81.2 |
| scaffold_1885 | + | 747 | 2889 | 2143 | 98.1 |
| scaffold_1885 | + | 2907 | 4452 | 1546 | 99.6 |
| scaffold_2527 | - | 1235 | 1959 | 725 | 99.5 |
| scaffold_3913 | - | 5196 | 5565 | 370 | 99.6 |
| scaffold_3958 | - | 1 | 2566 | 2566 | 80.0 |
| scaffold_4193 ^$^ | - | 14110 | 16792 | 2683 | 99.7 |
| scaffold_4268 | + | 408 | 1421 | 1014 | 99.6 |
| scaffold_4281 | + | 85 | 487 | 410 | 96.2 |
| scaffold_4387 | - | 1 | 1916 | 1916 | 97.5 |
| scaffold_4387 | - | 4990 | 5945 | 956 | 99.6 |
| scaffold_4387 | - | 4296 | 4797 | 502 | 99.6 |
| scaffold_6463 | + | 3655 | 6038 | 2384 | 99.6 |
| scaffold_6473 | - | 8640521 | 8642413 | 1901 | 97.7 |
| scaffold_6473 | - | 8638492 | 8640109 | 1618 | 99.7 |
| scaffold_6482 | + | 2243697 | 2245074 | 1378 | 91.5 |
| scaffold_6482 ^$^ | - | 1037675 | 1041750 | 4076 | 99.6 |
| scaffold_6482 ^$^ | + | 24246 | 26230 | 1985 | 93.0 |
| scaffold_6482 ^*^ | + | 2246673 | 2248739 | 2067 | 94.4 |
| scaffold_6482 ^$^ | + | 2289749 | 2290747 | 999 | 80.0 |
| scaffold_6496 | + | 110298 | 114367 | 4070 | 99.7 |
| scaffold_6496 | + | 24408367 | 24410430 | 2064 | 99.6 |
| scaffold_6498 ^$^ | - | 346406 | 349650 | 3245 | 94.0 |
| scaffold_6500 | - | 2822182 | 2826675 | 4493 | 99.7 |
| scaffold_6500 | - | 15064631 | 15064735 | 105 | 99.0 |
| scaffold_6500 | - | 27800534 | 27800865 | 332 | 80.0 |
| scaffold_6500 | - | 27799248 | 27799398 | 151 | 84.8 |
| scaffold_6500 ^$^ | + | 29721803 | 29723909 | 2106 | 93.2 |
| scaffold_6500 ^$^ | - | 27799630 | 27800328 | 699 | 80.0 |
| scaffold_6500 ^*^ | - | 28258012 | 28258571 | 560 | 80.0 |
| scaffold_6501 ^$^ | + | 79374 | 81668 | 2295 | 80.5 |
| **scaffold_6540 ^§^** | **+** | **6787901** | **6792402** | **4502** | **-** |
| scaffold_6540 | + | 9425297 | 9425488 | 192 | 100 |
| scaffold_6540 | + | 30770461 | 30770884 | 424 | 99.8 |
| scaffold_6541 | - | 1265178 | 1268930 | 3753 | 97.2 |
| scaffold_6541 | - | 1116909 | 1117300 | 392 | 96.2 |
| scaffold_6541^*^ | + | 1321376 | 1322010 | 635 | 98.1 |
| scaffold_6541 ^$^ | - | 1120980 | 1124220 | 3241 | 96.3 |

^§^ the reference *Helena* copy

* sequences with internal deletions and insertions

$ sequences with internal deletions
