# Supplementary figures and images for: The evolutionary dynamics of the Helena retrotransposon revealed by sequenced Drosophila genomes
Source: BMC Evol Biol. 2009 Jul 22;9:174. doi: 10.1186/1471-2148-9-174 (PMC3087515; doi:10.1186/1471-2148-9-174)

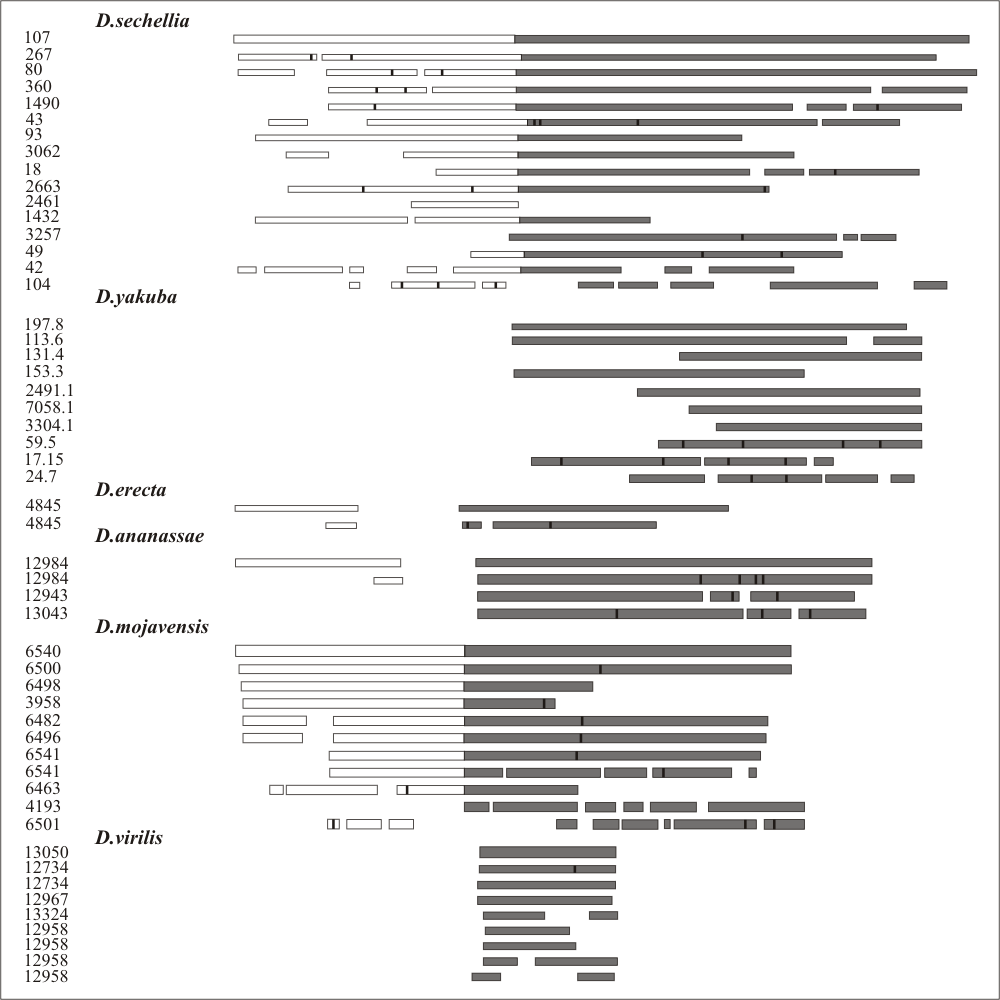

Supplement: Additional file 8 — Schematic representation of Helena. Schematic representation of Helena copies in D. sechellia, D. yakuba, D. erecta, D. ananassae, D. mojavensis and D. virilis. The sequences represented have at least 90% identity and 50% of the length of the reference copy, and with e-values of less than 10e-10. Spaces = indels. The first schematic representation is the reference copy in each species. White = gag. Gray = RTase. [file 1471-2148-9-174-S8.tiff]

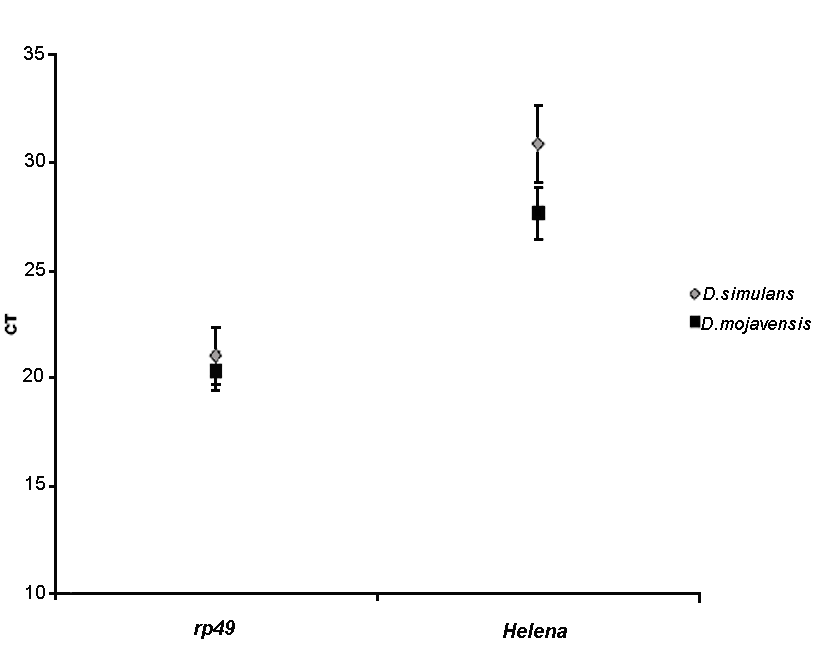

Supplement: Additional file 10 — Mean CT of the rp49 control gene and Helena. Ct comparison between rp49 (reference) and Helena real time PCR assays of D. simulans and D. mojavensis. Gray = mean CT of D. simulans. Black = mean CT of D. mojavensis. Std = standard deviation. [file 1471-2148-9-174-S10.tiff]
