# Supplementary material for: The evolutionary dynamics of the Helena retrotransposon revealed by sequenced Drosophila genomes
Source: BMC Evol Biol. 2009 Jul 22;9:174. doi: 10.1186/1471-2148-9-174 (PMC3087515; doi:10.1186/1471-2148-9-174)
Supplement: Additional file 9 — Species and strains, geographic origin and year of collection. The data provided is a list of D. simulans and D. mojavensis used in this study for analysis of Helena activity in natural populations. [file 1471-2148-9-174-S9.doc]

**Additional File 9.** Species and strains, geographic origin and year of collection.

| **Species** | **Location** | **Stock Number** | **Date** |
| --- | --- | --- | --- |
| *D. simulans* | North America, U.S.A* | 14021-0251.195 | 1991 |
|  | Junco do Serido, PB, Brazil |  | 2008 |
|  | Lençóis, BA, Brazil |  | 2005 |
|  | Itaúnas, ES, Brazil |  | 2005 |
|  | Onda Verde, SP, Brazil |  | 2005 |
|  | Ratones, SC, Brazil |  | 2005 |
|  | Seychelles |  |  |
|  | Winters, California, U.S.A* | 14021-0251.194 | 1995 |
|  | Noumea, New Caledonia* | 14021-0251.198 | 1991 |
|  | New Caledonia, Scotland* | 14021-0251.216 | 1991 |
|  | Amieu, France |  |  |
|  | Valence, France |  |  |
| *D. mojavensis* | Grand Canyon, Arizona, U.S.A |  |  |
|  | Catalina Island, California, U.S.A | 15081-1352.02 | 1991 |
|  | Santa Rosa Mountains, Arizona, U.S.A | 15081-1352.09 | 1993 |
|  | Catalina Island, California, U.S.A* | 15081-1352.22 | 2002 |
|  | Sonora, Mexico | 15081-1352.24 | 2003 |

* Strains with the genome sequenced
